# Bufalin Suppresses Pancreatic Ductal Adenocarcinoma Through ER Stress-Ferroptosis Crosstalk Associated with IP_3_R-Linked Ca^2+^ Dysregulation and ATF3/SLC7A11 Regulation

**DOI:** 10.3390/ijms27104373

**Published:** 2026-05-14

**Authors:** Pei-Wen Yang, Xin Li, Wai-Mei Si, Yuan Zhang, Xiang-Yu Kong, Xin-Yi Xu, Xiao-Yan Zhu, Zhen Chen

**Affiliations:** 1 Department of Integrative Oncology, Shanghai Cancer Center, Fudan University, Shanghai 200032, China; 2 Department of Oncology, Shanghai Medical College, Fudan University, Shanghai 200032, China

**Keywords:** bufalin, pancreatic ductal adenocarcinoma, ferroptosis, ER stress, IP_3_R, ATF3

## Abstract

Pancreatic ductal adenocarcinoma (PDAC) is a highly aggressive malignancy characterized by therapeutic resistance and poor prognosis, underscoring the need for new therapeutic strategies. Bufalin, a major bioactive constituent of *Venenum bufonis*, has shown antitumor activity in several cancer types; however, its mechanism of action in PDAC remains incompletely defined. In this study, we investigated the antitumor effects of bufalin in PDAC using *in vitro* assays, mouse tumor models, and integrative transcriptomic, proteomic, metabolomic, and bioinformatic analyses. Bufalin inhibited PDAC cell viability, clonogenic growth, migration, and tumor progression *in vivo*. Pharmacological rescue experiments indicated that ferroptosis contributes importantly to bufalin-induced cytotoxicity, although apoptosis- and pyroptosis-related pathways may also be involved. Multi-omics analyses revealed coordinated alterations in calcium homeostasis, endoplasmic reticulum (ER) stress/unfolded protein response (UPR) signaling, and ferroptosis-related metabolic pathways. Further experiments showed that bufalin was associated with disrupted intracellular Ca^2+^ homeostasis, IP_3_R-linked ER Ca^2+^ release, activation of PERK/eIF2α/ATF4 signaling, increased ATF3 expression, reduced SLC7A11 and GPX4 expression, glutathione depletion, and enhanced lipid peroxidation. Molecular docking and surface plasmon resonance assays supported an *in vitro* physical interaction between bufalin and IP_3_R1/IP_3_R3, while inhibition of ER stress attenuated several bufalin-induced ferroptosis-related phenotypes. Bioinformatic analyses further showed that higher ER stress and ferroptosis signature scores were associated with improved overall survival in PDAC, and concurrent activation of both signatures was linked to the most favorable prognosis. Collectively, these findings support that bufalin suppresses PDAC progression through coordinated ER stress- and ferroptosis-related responses, highlighting ER stress-ferroptosis crosstalk as a potential therapeutic vulnerability in PDAC.

## 1. Introduction

Pancreatic ductal adenocarcinoma (PDAC) exhibits aggressive biological behavior and limited responsiveness to current therapeutic interventions, resulting in a five-year survival rate of less than 10% [1,2,3,4,5,6,7]. The predominance of advanced-stage presentation, coupled with aggressive tumor phenotypes and inherent resistance to conventional regimens, substantially restricts treatment effectiveness. Therefore, identifying novel pharmacological strategies that target intrinsic vulnerabilities in PDAC cells and improve therapeutic responses remains an urgent clinical priority.

*Venenum bufonis*, prepared from the dried glandular secretions of *Bufo bufo gargarizans Cantor* or *Bufo melanostictus Schneider*, has long been incorporated into East Asian medical practice for the management of inflammatory conditions and tumor-related diseases [8,9]. Bufalin, a major cardiotonic bufadienolide isolated from *Venenum bufonis*, exhibits broad-spectrum antitumor activity. Previous studies have shown that bufalin suppresses tumor cell proliferation and migration and induces multiple forms of regulated cell death across diverse cancer types [10,11,12,13,14,15,16,17,18,19,20,21,22]. However, the pharmacological basis underlying bufalin activity in PDAC and its mechanistic linkage to specific regulated cell death pathways remain incompletely defined, limiting its rational development as an ethnomedicine-derived anticancer candidate.

Ferroptosis represents a distinct mode of regulated cell death driven by iron-dependent accumulation of lipid peroxides and impairment of the system Xc^−^-glutathione (GSH)-glutathione peroxidase 4 (GPX4) antioxidant defense pathway [23,24,25,26]. Increasing evidence indicates that ferroptosis induction can suppress tumor growth and may overcome resistance to chemotherapy, radiotherapy or immunotherapy [27,28,29,30,31,32]. For example, inhibition of system Xc^−^ by erastin leads to cysteine depletion, impaired GSH synthesis, reduced GPX4 activity, and subsequent ferroptosis [26,33]. Given the metabolic reprogramming and elevated oxidative stress burden in PDAC, ferroptosis has emerged as a potential therapeutic vulnerability.

The endoplasmic reticulum (ER) functions as a central organelle responsible for protein maturation, lipid biosynthesis, and regulation of intracellular calcium dynamics. ER stress activates the unfolded protein response (UPR) through three principal signaling branches mediated by PERK, ATF6, and IRE1α [34,35,36,37]. While transient UPR activation restores ER homeostasis, sustained ER stress can trigger cell death programs. Notably, recent studies have revealed functional crosstalk between ER stress signaling and ferroptosis via regulation of lipid metabolism and redox balance, including modulation of key ferroptosis-related genes such as SLC7A11 and GPX4 [38,39,40,41,42,43]. However, the causal relationships among ER calcium disturbance, UPR activation, and ferroptosis-related responses may vary across cellular contexts and require careful experimental validation.

Nevertheless, whether and how bufalin suppresses PDAC progression through ER stress-ferroptosis crosstalk remains incompletely understood. In the present study, we evaluated the antitumor effects of bufalin in PDAC using *in vitro* functional assays and murine tumor models, and further performed integrated transcriptomic, proteomic, and metabolomic analyses to characterize the molecular networks altered by bufalin treatment. In particular, we examined the relationship among ER calcium homeostasis, UPR signaling, and ferroptosis-related regulation, with emphasis on IP_3_R-associated Ca^2+^ dysregulation and PERK/ATF3/SLC7A11/GPX4-related changes. These data support that bufalin suppresses PDAC progression through coordinated ER stress- and ferroptosis-related responses.

## 2. Results

### 2.1. Bufalin Suppresses Pancreatic Cancer In Vivo and In Vitro

**Figure 1:**
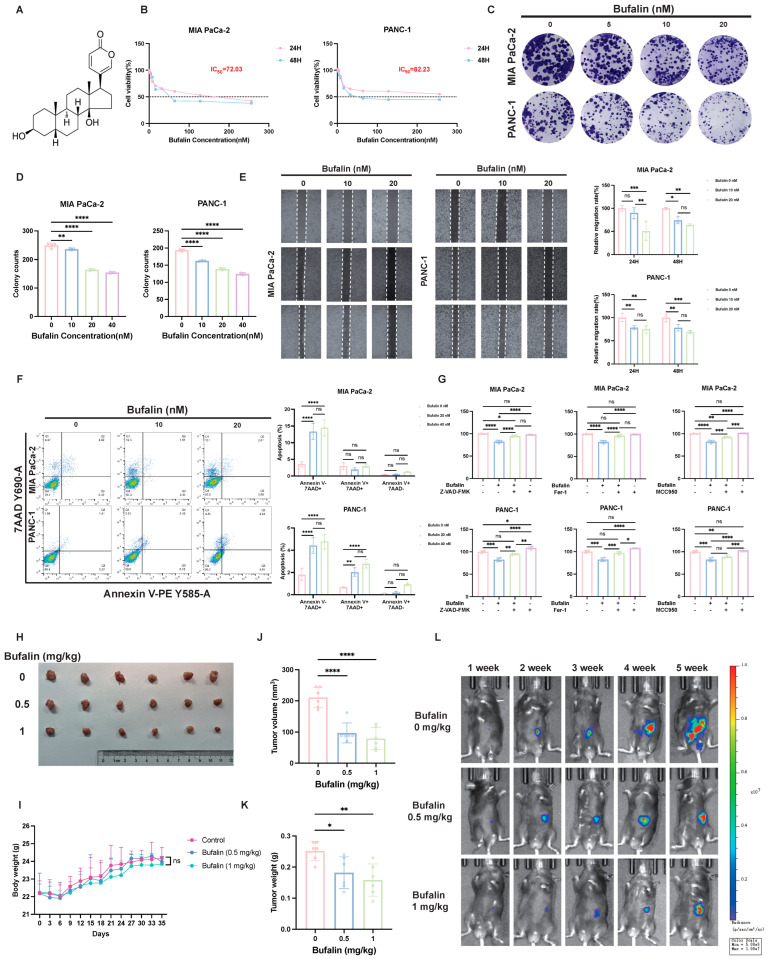
Bufalin suppresses pancreatic cancer growth *in vitro* and *in vivo* through inhibition of proliferation and migration and induction of multiple cell death-related responses. (**A**) Chemical structure of bufalin. (**B**) CCK-8 assay showing bufalin reduced the viability of MIA PaCa-2 and PANC-1 cells in a dose- and time-dependent manner (24/48 h treatment); IC_50_ values at 48 h were 72.03 nM (MIA PaCa-2) and 82.23 nM (PANC-1). (**C**,**D**) Colony formation assay demonstrating significantly diminished colony-forming capacity of MIA PaCa-2 and PANC-1 cells following 14-day treatment with bufalin (5, 10, 20 nM). (**E**) Wound-healing assay showing bufalin inhibited the migratory capacity of MIA PaCa-2 and PANC-1 cells, as reflected by reduced wound closure. (**F**) Annexin V-PE/7AAD staining revealing bufalin treatment increased the proportion of necrotic cells in PDAC cells. (**G**) CCK-8 assay to identify predominant cell death modalities: MIA PaCa-2 and PANC-1 cells were treated with bufalin (20 nM) alone or in combination with apoptosis inhibitor Z-VAD-FMK (20 μM), pyroptosis inhibitor MCC950 (10 μM), or ferroptosis inhibitor Fer-1 (2.5 μM) for 48 h. (**H**) Representative images of tumors harvested at the end of the experiment, all inhibitors partially reversed bufalin-induced growth suppression, indicating that ferroptosis contributes to bufalin-induced cytotoxicity, while apoptosis- and pyroptosis-related pathways may also participate. (**I**) Body weights from each experimental group (*n* = 6 per group). (**J**) Final tumor volume from each experimental group (*n* = 6 per group). (**K**) Final tumor weights from each experimental group (*n* = 6 per group). (**L**) Representative bioluminescent images showing tumor burden in mice from different groups. All data are expressed as mean ± SD (* *p* < 0.05; ** *p* < 0.01; *** *p* < 0.001; **** *p* < 0.0001).

To initially assess the antitumor activity of bufalin in pancreatic cancer, we examined its effects on the proliferation of MIA PaCa-2, PANC-1, AsPC-1 cells, and BxPC-3 using the CCK-8 assay. Bufalin inhibited cell viability in both a dose- and time-dependent manner after Bufalin treatment (Figure 1A,B and Figure S1A,B). The 48 h IC_50_ values were 72.03 nM for MIA PaCa-2 cells, 82.23 nM for PANC-1 cells, 7.8 nM for AsPC-1 cells, and 37.29 nM for BxPC-3 cells. Based on these results, 5, 10, and 20, 40 nM were selected for subsequent *in vitro* experiments in MIA PaCa-2 and PANC-1 cells. Consistent with these findings, colony formation assays showed that bufalin markedly reduced the long-term clonogenic capacity of both cell lines (Figure 1C,D). Wound-healing assays further indicated that bufalin impaired migratory ability (Figure 1E). Annexin V-PE/7AAD staining showed that bufalin increased the proportion of dead cells in both cell lines after 48 h of exposure (Figure 1F). To preliminarily characterize the mode of cell death induced by bufalin, cells were treated with different concentration of bufalin alone or together with Z-VAD-FMK, MCC950, or Fer-1, followed by viability analysis. At lower bufalin concentrations, three pharmacological inhibition partially attenuated bufalin-induced growth suppression, indicating the involvement of multiple cell death programs (Figure 1G). However, when bufalin was increased to near-IC_50_ concentrations, all three inhibitors showed only limited rescue effects (Figure S1C–F), suggesting that stronger bufalin-induced cytotoxicity is not governed by a single dominant pathway and may instead reflect the combined contribution of multiple regulated cell death mechanisms.

We next evaluated the antitumor effect of bufalin *in vivo* using an orthotopic murine pancreatic allograft model. Compared with the control group, bufalin treatment significantly reduced tumor volume and tumor weight (Figure 1H–L), supporting the antitumor activity of bufalin *in vivo*.

### 2.2. Integrative Multi-Omics Analyses Implicate Calcium Homeostasis, ER Stress, and Ferroptosis-Related Pathways in the Response to Bufalin

**Figure 2:**
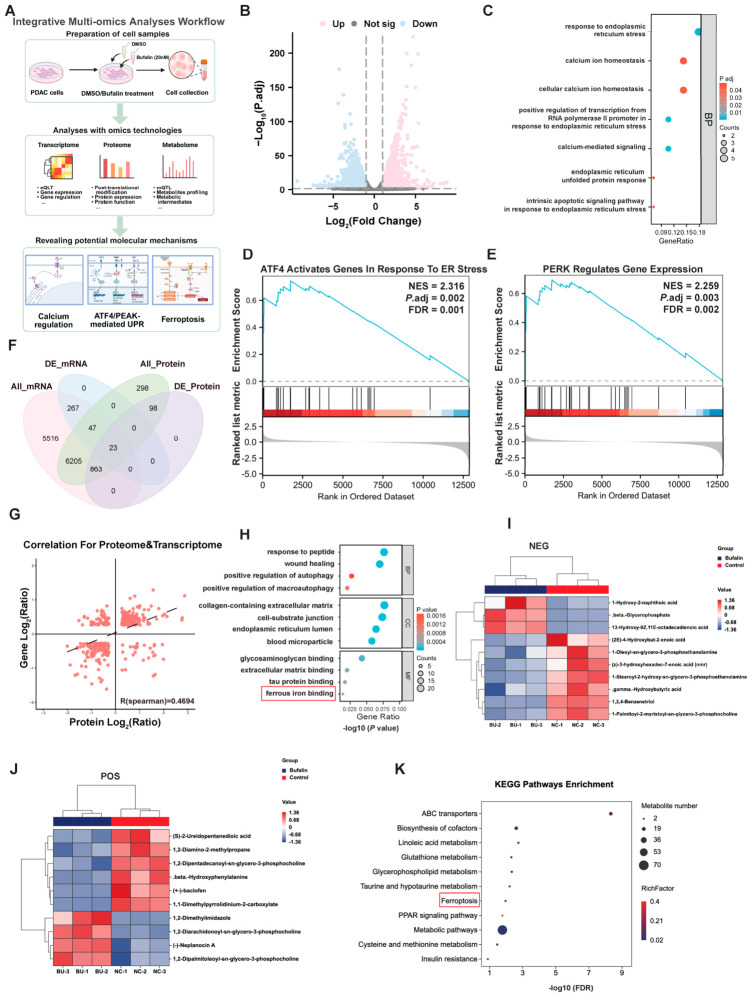
Integrative multi-omics analyses implicate ER stress- and ferroptosis-related pathways in the response to bufalin in PDAC. (**A**) Schematic diagram summarizing the potential mechanism of bufalin-mediated pancreatic cancer regulation, integrated from transcriptomic, proteomic, and metabolomic findings. (**B**) Volcano plot of RNA-seq data showing DEGs in bufalin-treated vs. control cells (|log_2_ fold change| > 1, *p* < 0.05). (**C**) GO enrichment analyses of upregulated DEGs. (**D**,**E**) GSEA showing enrichment of PERK pathway-regulated genes and ATF4-activated ER stress response in DEGs. (**F**) Venn diagrams showing the overlap of total and differential molecules between transcriptome and DIA proteome. (**G**) Spearman correlation analysis between mRNA and protein expression fold changes (R = 0.4694). (**H**) GO enrichment of differential proteins. (**I**,**J**) Cluster plots of differential metabolites identified by untargeted metabolomics under NEG and POS modes. (**K**) KEGG pathway enrichment analysis of differential metabolites.

To elucidate the molecular programs associated with the anticancer effects of bufalin in PDAC, we performed integrative multi-omics analyses combining RNA-seq, DIA-based proteomics, and untargeted metabolomics (Figure 2A). RNA-seq identified 12,669 mRNAs, including 337 differentially expressed genes after bufalin treatment (Figure 2B). Enrichment analysis showed that these genes were mainly associated with ER stress, UPR, PERK signaling, and calcium homeostasis (Figure 2C). Consistently, GSEA demonstrated enrichment of PERK pathway-regulated genes and ATF4-responsive genes under ER stress conditions (Figure 2D,E). RNA-seq further showed altered expression of representative genes involved in the three canonical UPR branches, including increased expression of XBP1, HSPA5, ATF4, DDIT3/CHOP, PPP1CA, ATF3, CHAC1, TRIB1, and BCL2L1, together with decreased SEL1L expression (Figure S2A), supporting activation of the UPR program, particularly the PERK branch, in response to bufalin.

We next integrated the transcriptomic and proteomic datasets. DIA-based proteomics quantified 8036 proteins and identified 340 differentially expressed proteins (Figure S2B). Cross-omics comparison showed partial overlap between detected transcripts and proteins (Figure 2F), and Spearman correlation analysis revealed moderate concordance between mRNA and protein changes (R = 0.4694; Figure 2G). Functionally, proteomic enrichment analysis linked the differentially expressed proteins to pathways related to ferrous iron binding and cholesterol metabolism (Figure 2H). Although transcriptomic and proteomic alterations were only partially coupled at the individual molecule level, they converged on stress-response and metabolism-related pathways. Joint RNA-seq and proteomic GSEA further revealed pathway-level alterations in Ca^2+^ transport and PIP2 hydrolysis, strong enrichment of ATF4/PERK-associated ER stress and UPR programs, and remodeling of oxidative stress defense, iron-related redox regulation, and lipid metabolic pathways (Figure S2C). These convergent pathway signatures provided a rationale for subsequent functional analyses focused on Ca^2+^ dyshomeostasis, ER stress activation, and ferroptosis-related phenotypes.

Untargeted metabolomics further showed broad metabolic remodeling after bufalin treatment. Differential metabolites were identified in both negative and positive ion modes (Figure 2I,J and Figure S2D,E). KEGG enrichment analysis indicated that bufalin affected ferroptosis, glutathione metabolism, cysteine and methionine metabolism, and lipid-associated pathways, including glycerophospholipid metabolism, linoleic acid metabolism, and PPAR signaling (Figure 2K and Figure S2F). Together, these multi-omics results suggest that bufalin treatment is associated with coordinated changes in calcium homeostasis, ER stress/UPR signaling, oxidative stress defense, and ferroptosis-related metabolic programs. Importantly, rather than serving as standalone causal evidence, these multi-omics findings guided the subsequent validation experiments examining ER Ca^2+^ release, PERK/eIF2α/ATF4 activation, ATF3/SLC7A11 regulation, and ferroptosis-related biochemical changes in PDAC cells.

### 2.3. Bioinformatic Analyses Reveal the Correlation of Ferroptosis with ER Stress in PDAC

**Figure 3:**
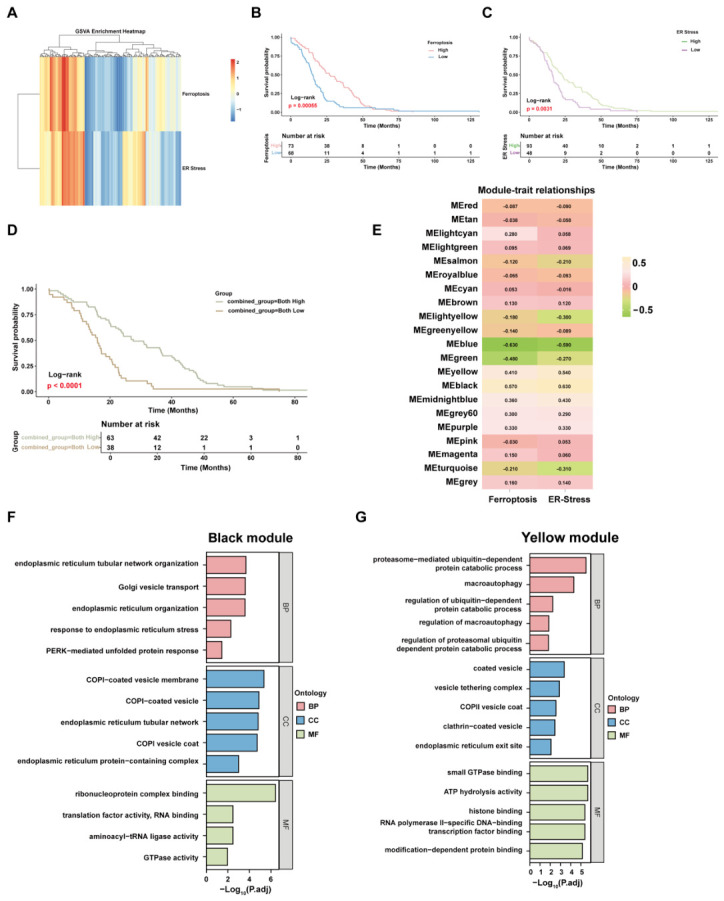
Bioinformatic analyses reveal coordinated activation of ER stress and ferroptosis in PDAC and their clinical relevance. (**A**) GSVA enrichment heatmap showing ferroptosis and ER stress pathway activity scores across PDAC samples. Each column represents an individual tumor sample, and rows represent pathway activity levels. (**B**) Kaplan-Meier survival analysis comparing overall survival between patients with high and low ferroptosis activity scores (log-rank test). (**C**) Kaplan-Meier survival analysis of overall survival based on ER stress activity scores (log-rank test). (**D**) Kaplan-Meier survival curves stratified by combined ferroptosis and ER stress activity scores. Patients with concurrent high activation of both pathways exhibited the most favorable prognosis (log-rank test). (**E**) Module-trait relationship heatmap generated by weighted gene co-expression network analysis, showing correlation coefficients between gene modules and ferroptosis or ER stress scores. The black module displayed the strongest positive correlation with both pathways. (**F**) GO enrichment analysis of genes within the black module, revealing significant enrichment in endoplasmic reticulum stress response, UPR, and PERK-mediated UPR pathways. (**G**) GO enrichment analysis of genes within the yellow module, showing enrichment in proteasome- and autophagy-related processes, indicating pathway specificity.

To determine whether ER stress and ferroptosis are associated at the clinical and systems level in PDAC, we analyzed transcriptomic and survival datasets. GSVA showed partially overlapping activation patterns of ferroptosis- and ER stress-related signatures across tumor samples (Figure 3A). Kaplan-Meier analysis indicated that higher ferroptosis activity and higher ER stress activity was each associated with improved overall survival (Figure 3B,C). Patients with concurrent high ferroptosis and high ER stress scores showed the most favorable prognosis (Figure 3D).

We next performed weighted gene co-expression network analysis to identify modules associated with both signatures. The black module showed the strongest positive correlation with ferroptosis and ER stress scores (Figure 3E). Functional enrichment analysis of this module highlighted ER stress response, UPR, and PERK-related signaling terms (Figure 3F), whereas another representative module showed enrichment in proteasome- and autophagy-related processes (Figure 3G). These analyses support a transcriptional association between ER stress and ferroptosis in PDAC and provide a systems-level rationale for the subsequent functional validation experiments.

### 2.4. Bufalin Induces ER Stress and Ferroptosis In Vitro and In Vivo

**Figure 4:**
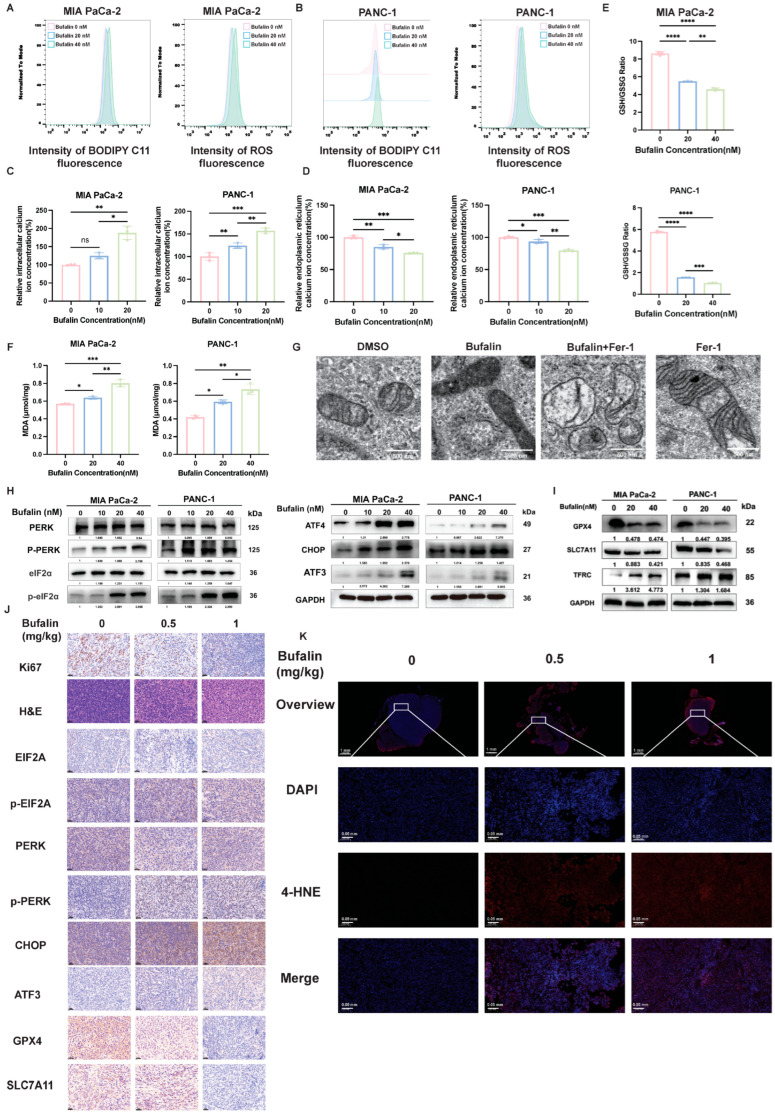
Bufalin induces ferroptosis and ER stress in PDAC. (**A**,**B**) Flow cytometry analysis of ferroptosis-related oxidative stress markers: (**A**) lipid peroxides (detected by BODIPY C11 probe) and (**B**) total ROS (detected by DCFH-DA probe) in MIA PaCa-2 and PANC-1 cells after bufalin treatment. (**C**,**D**) Quantification of (**C**) cytosolic calcium concentrations, and (**D**) ER (reflecting calcium dyshomeostasis) in bufalin-treated MIA PaCa-2 and PANC-1 cells. (**E**,**F**) Colorimetric assay quantification of ferroptosis-related biochemical markers in MIA PaCa-2 and PANC-1 cells treated with 0, 20, or 40 nM bufalin for 48 h: (**E**) with reduced GSH/GSSG ratio and (**F**) MDA levels. (**G**) Representative TEM images of MIA PaCa-2 and PANC-1 cells treated with bufalin (20 nM) alone or in combination with the ferroptosis inhibitor Fer-1 (2.5 μM) for 48 h. (**H**) Western blot analysis of ER stress-related proteins in the PERK/eIF2α/ATF4 pathway: p-PERK, total PERK, p-eIF2α, total eIF2α, ATF4, CHOP, and ATF3 in MIA PaCa-2 and PANC-1 cells treated with 0, 10, 20, or 40 nM bufalin for 48 h. (**I**) Western blotting analysis of ferroptosis-related proteins: GPX4, SLC7A11, ATF3, and TFRC in MIA PaCa-2 and PANC-1 cells treated with 0, 20, or 40 nM bufalin for 48 h. (**J**) Representative H&E staining and IHC staining of tumor tissues (from orthotopic pancreatic allograft models) for Ki67, ER stress-related proteins (p-PERK, PERK, p-EIF2A, EIF2A, CHOP, ATF3), and ferroptosis-related proteins (SLC7A11, GPX4). Scale bar = 25 μm. (**K**) Representative immunofluorescence images of 4-HNE (red, lipid peroxidation marker) in tumor tissues from mice treated with vehicle (0 mg/kg) or bufalin (0.5, 1 mg/kg). Scale bar = 0.05 mm. All data are expressed as mean ± SD (* *p* < 0.05; ** *p* < 0.01; *** *p* < 0.001; **** *p* < 0.0001).

Based on the convergent multi-omics and bioinformatic findings described above, we next performed functional validation experiments to determine whether bufalin induces Ca^2+^ dyshomeostasis, ER stress activation, and ferroptosis-related phenotypes in PDAC cells. Bufalin significantly increased lipid peroxidation and total ROS levels, as measured by BODIPY C11 and DCFH-DA staining, respectively (Figure 4A,B and Figure S3A,B). In parallel, bufalin increased cytosolic Ca^2+^ and reduced ER Ca^2+^ levels (Figure 4C,D), indicating disruption of intracellular calcium homeostasis. Moreover, bufalin decreased the GSH/GSSG ratio and increased MDA levels (Figure 4E,F). Transmission electron microscopy revealed mitochondrial shrinkage and increased membrane density in bufalin-treated cells (Figure 4F and Figure S3C,D). At the protein level, bufalin increased p-PERK/PERK and p-eIF2α/eIF2α ratios and upregulated ATF4, CHOP, and ATF3 (Figure 4H and Figure S3F), consistent with activation of a PERK-associated ER stress response. Western blotting further showed reduced GPX4 and SLC7A11 expression together with increased TFRC and ATF3 expression (Figure 4I and Figure S3G). Consistent with the *in vitro* findings, immunohistochemical staining of tumor tissues showed decreased Ki67, SLC7A11, and GPX4 expression and increased staining for p-PERK, p-eIF2α, CHOP, and ATF3 in bufalin-treated tumors (Figure 4J). Immunofluorescence assay found Bufalin treatment significantly increased 4-HNE intensity in PDAC xenografts in a dose-dependent manner, indicating enhanced lipid peroxidation *in vivo* (Figure 4K). Overall, these data indicate that bufalin treatment is associated with Ca^2+^ dyshomeostasis, ER stress-associated signaling, and ferroptosis-related biochemical and morphological changes in PDAC models.

### 2.5. IP_3_R-Associated ER Ca^2+^ Release and ER Stress Are Implicated in Bufalin-Induced Ferroptosis-Related Changes

**Figure 5:**
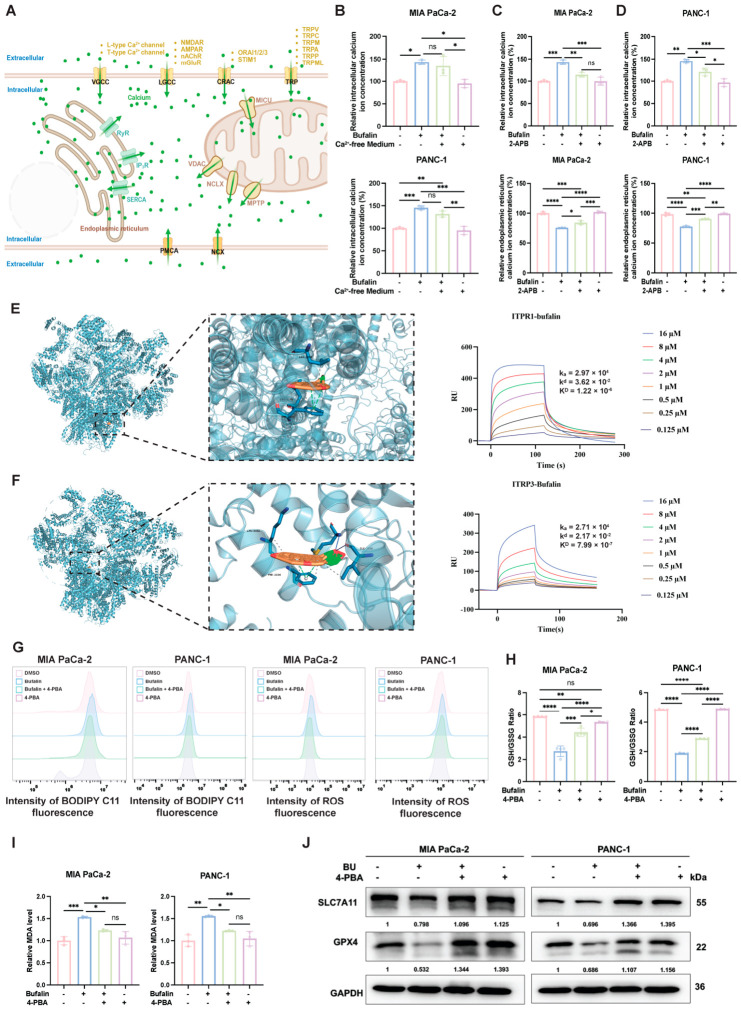
Bufalin interacts with IP_3_R and promotes ER stress-associated ferroptotic responses in PDAC cells. (**A**) Schematic diagram illustrating the key calcium channels and transporters involved in intracellular calcium homeostasis, including plasma membrane channels (VGCC, CRAC, TRP), ER-resident channels (RyR, IP3R, and SERCA), and mitochondrial calcium regulators (MCU, VDAC, NCLX). (**B**) Intracellular calcium concentration in MIA PaCa-2 and PANC-1 cells treated with bufalin in the presence or absence of calcium-free medium. (**C**,**D**) Intracellular calcium concentration and ER calcium content in MIA PaCa-2 (**C**) and PANC-1 (**D**) cells treated with bufalin alone or in combination with the IP_3_R inhibitor 2-APB. (**E**,**F**) Molecular docking and surface plasmon resonance (SPR) analysis of bufalin binding to IP_3_R1 (**E**) and IP_3_R3 (**F**). The left panel shows the predicted binding mode, and the right panel displays the SPR sensorgrams and calculated binding affinities (KD values). (**G**) Flow cytometry analysis of lipid peroxidation and ROS levels in PDAC cells treated with bufalin alone or with the ER stress inhibitor 4-PBA. (**H**,**I**) Quantification of the GSH/GSSG ratio and MDA levels in PDAC cells after the indicated treatments, showing that 4-PBA partially reversed bufalin-induced redox imbalance and lipid peroxidation. (**J**) Western blot analysis of SLC7A11 and GPX4 expression in MIA PaCa-2 and PANC-1 cells following treatment with bufalin and/or 4-PBA. GAPDH was used as the loading control. All data are expressed as mean ± SD (* *p* < 0.05; ** *p* < 0.01; *** *p* < 0.001; **** *p* < 0.0001).

Given that the multi-omics analysis revealed alterations in calcium homeostasis- and ER stress-related pathways, and that IP_3_Rs and RyRs are major ER calcium-release channels (Figure 5A), we next explored the potential source of bufalin-induced cytosolic Ca^2+^ elevation. Cells were treated with bufalin in calcium-free medium to distinguish intracellular Ca^2+^ release from extracellular Ca^2+^ influx. Under these conditions, the bufalin-induced increase in cytosolic Ca^2+^ was not markedly altered (Figure 5B), suggesting that intracellular Ca^2+^ stores contribute substantially to this response.

We then examined the possible involvement of major ER calcium-release channels using pharmacological inhibitors. DAN reduced cytosolic Ca^2+^ levels in MIA PaCa-2 cells but showed no obvious effect in PANC-1 cells and did not significantly restore ER Ca^2+^ levels in either cell line (Figure S3A). In contrast, 2-APB attenuated the bufalin-induced increase in cytosolic Ca^2+^ and partially restored ER Ca^2+^ levels (Figure 5C,D). These findings are consistent with the involvement of IP_3_R-associated ER Ca^2+^ release in the cellular response to bufalin, although genetic validation will be required to more definitively establish the specific contribution of IP_3_R isoforms.

To further assess whether bufalin may interact with IP_3_R proteins, molecular docking analysis was performed and predicted favorable binding of bufalin to IP_3_R1 and IP_3_R3, with binding energies of −8.3 and −7.4 kcal·mol^−1^, respectively. In addition, SPR assays showed concentration-dependent binding of bufalin to recombinant IP_3_R1 and IP_3_R3 proteins in vitro (Figure 5E,F). These results support a potential physical interaction between bufalin and IP_3_R1/IP_3_R3, but do not by themselves establish the functional requirement of IP_3_R in intact cells.

Because ER Ca^2+^ depletion can trigger ER stress, we next used the ER stress inhibitor 4-PBA to determine whether ER stress inhibition could attenuate bufalin-induced ferroptosis-related phenotypes. Co-treatment with 4-PBA reduced bufalin-induced ROS accumulation and lipid peroxidation (Figure 5G and Figure S4B,C), restored the decreased GSH/GSSG ratio, and reduced MDA levels (Figure 5H,I). Consistently, Western blot analysis showed that 4-PBA partially reversed bufalin-induced downregulation of SLC7A11 and GPX4 (Figure 5J and Figure S4D). Together, these findings support an association among bufalin-induced ER Ca^2+^ dysregulation, ER stress activation, and ferroptosis-related changes in PDAC cells.

### 2.6. ATF3 Contributes to Bufalin-Induced Ferroptosis-Related Changes Through Regulation of SLC7A11

**Figure 6:**
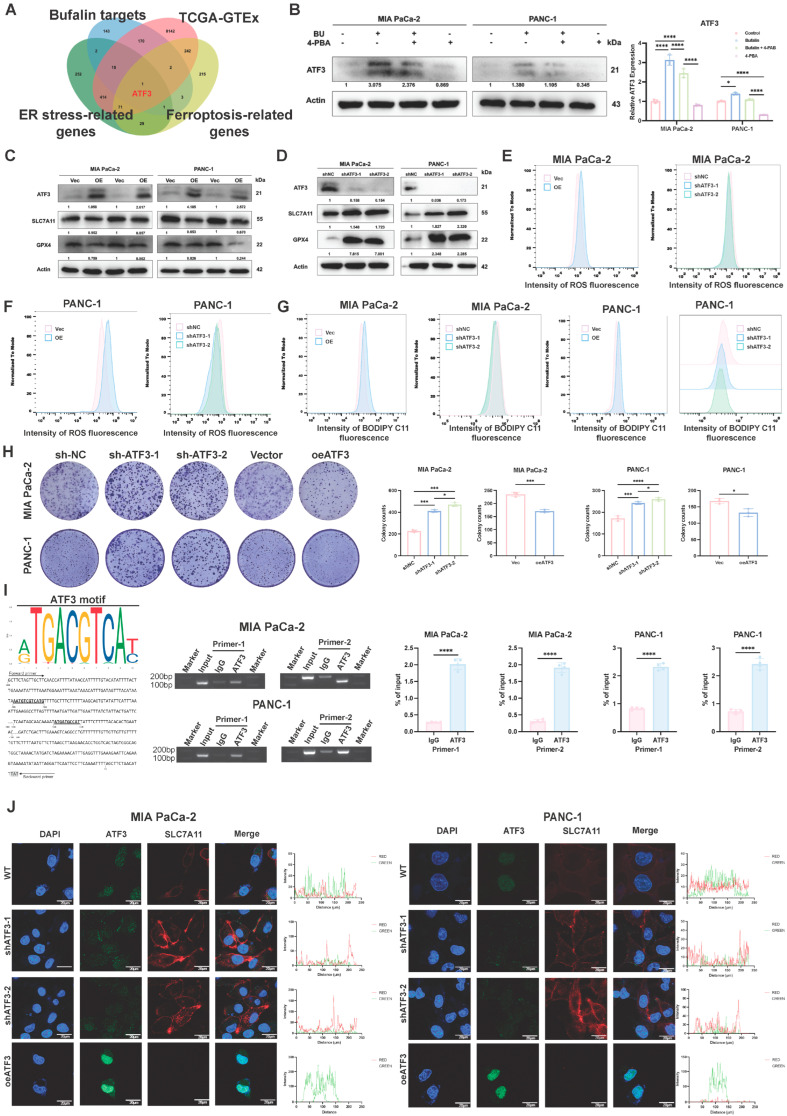
ATF3 mediates the link between bufalin-induced ER stress and ferroptosis in PDAC cells. (**A**) Venn diagram showing the intersection of bufalin targets, PDAC DEGs, ER stress-related genes, and ferroptosis-related genes; ATF3 was the only overlapping gene. (**B**) Western blot analysis of ATF3 expression in cells co-treated with bufalin and 4-PBA for 48 h; 4-PBA reversed bufalin-induced ATF3 upregulation. (**C**,**D**) Western blot analysis of SLC7A11 and GPX4 in stable ATF3 overexpression (**C**) and knockdown (**D**) MIA PaCa-2/PANC-1 cells; ATF3 overexpression downregulated, while knockdown upregulated, both proteins. (**E**–**G**) Flow cytometry analysis of ROS (**E**,**F**) and lipid peroxidation (**G**) levels in ATF3-overexpressing or ATF3-knockdown MIA PaCa-2 and PANC-1 cells. (**H**) Colony formation assay showing the proliferative capacity of PDAC cells following ATF3 overexpression or knockdown. (**I**) Chromatin immunoprecipitation (ChIP) assay demonstrating direct binding of ATF3 to the SLC7A11 promoter. Left: Consensus ATF3 binding motif and corresponding sequences in the SLC7A11 promoter, with primer locations indicated. Middle: Agarose gel electrophoresis of ChIP-PCR products using two independent primer pairs. Right: Quantitative ChIP-qPCR results showing ATF3 enrichment at the promoter, presented as % of input. (**J**) Immunofluorescence staining of ATF3 (green) and SLC7A11 (red) in MIA PaCa-2 and PANC-1 cells with ATF3 knockdown or overexpression. Nuclei were counterstained with DAPI (blue). Fluorescence intensity profiles are shown on the right. Scale bars = 20 μm. All data are expressed as mean ± SD (* *p* < 0.05; *** *p* < 0.001; **** *p* < 0.0001).

To identify downstream effectors connecting ER stress to ferroptosis-related regulation, we intersected bufalin targets, PDAC DEGs, ER stress-related genes, and ferroptosis-related genes. ATF3 emerged as the only overlapping candidate (Figure 6A). Notably, the ER stress inhibitor 4-PBA reduced bufalin-induced ATF3 upregulation (Figure 6B).

To examine its functional role, we generated ATF3 knockdown and overexpression models. ATF3 overexpression reduced SLC7A11 and GPX4 protein levels, whereas ATF3 knockdown increased their expression (Figure 6C,D and Figure S5A,B). Likewise, ATF3 overexpression increased ROS and lipid peroxidation, while ATF3 knockdown had the opposite effect (Figure 6E–G and Figure S5C,D). Colony formation assays showed that ATF3 overexpression impaired clonogenic growth, whereas ATF3 silencing promoted it (Figure 6H).

We next investigated whether ATF3 directly regulates SLC7A11 transcription. Chip assays showed that ATF3 was enriched at the predicted binding region within the SLC7A11 promoter in both MIA PaCa-2 and PANC-1 cells compared with IgG controls (Figure 6I). To further test the functional relevance of this binding site, we performed dual-luciferase reporter assays using wild-type and mutant SLC7A11 promoter constructs. ATF3 overexpression significantly reduced the activity of the wild-type SLC7A11 promoter, whereas mutation of the predicted ATF3-binding motif largely abolished this repressive effect (Figure S5E). These results provide functional evidence that ATF3 can repress SLC7A11 promoter activity through the predicted binding site.

Furthermore, immunofluorescence analysis showed reciprocal expression patterns of ATF3 and SLC7A11 in ATF3-overexpressing and ATF3-knockdown cells (Figure 6J). Together, these findings support a model in which ATF3 contributes to bufalin-induced ferroptosis-related changes, at least in part, by transcriptionally repressing SLC7A11 and thereby weakening the SLC7A11/GPX4 antioxidant defense axis.

### 2.7. Ferroptosis Contributes Importantly to Bufalin’s Anticancer Activity in PDAC In Vitro and In Vivo

**Figure 7:**
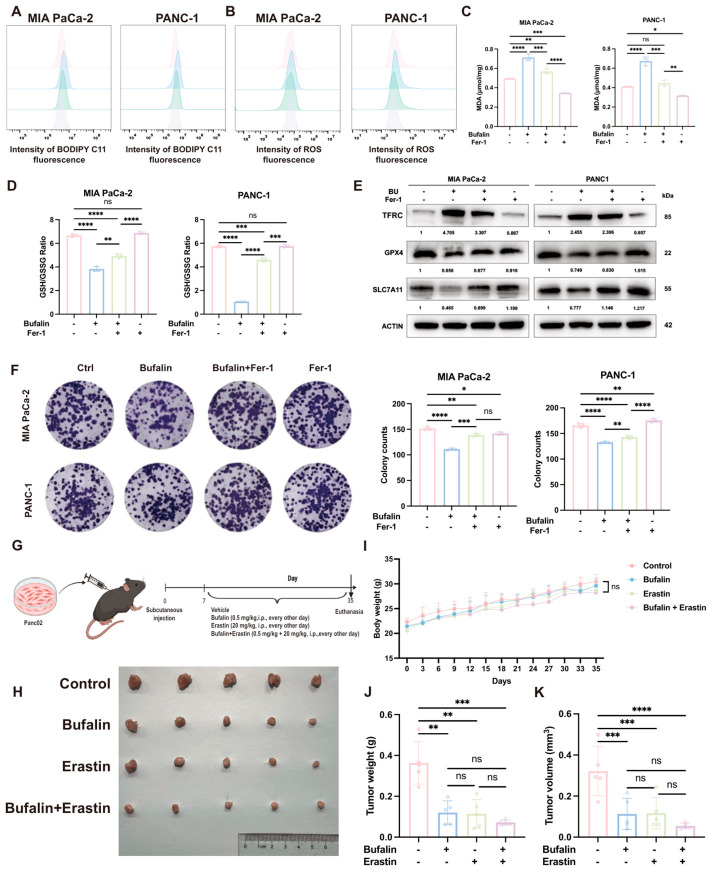
Ferroptosis contributes to bufalin’s anticancer activity in PDAC *in vitro* and *in vivo*. (**A**,**B**) Flow cytometry analysis of ferroptosis-related oxidative stress markers in MIA PaCa-2 and PANC-1 cells co-treated with bufalin and Fer-1: (**A**) lipid peroxides and (**B**) ROS; Fer-1 significantly reversed bufalin-induced elevations in both markers. (**C**,**D**) Colorimetric assay quantification of ferroptosis-related biochemical indices in cells co-treated with bufalin and Fer-1 for 48 h: (**C**) MDA levels were reduced by Fer-1, and (**D**) GSH/GSSG ratio (suppressed by bufalin) was restored by Fer-1. (**E**) Western blot analysis of ferroptosis-related proteins (TFRC, GPX4, SLC7A11) in cells co-treated with bufalin and Fer-1 for 48 h; Fer-1 partially reversed bufalin-mediated upregulation of TFRC and downregulation of GPX4/SLC7A11. (**F**) Colony formation assay showing the effect of Fer-1 on bufalin-mediated growth inhibition: MIA PaCa-2 and PANC-1 cells were co-treated with bufalin and Fer-1 for 14 days; Fer-1 partially rescued the bufalin-induced reduction in colony-forming capacity. (**G**) Schematic diagram of the *in vivo* experiment: mouse subcutaneous xenograft models of pancreatic cancer were established, followed by intraperitoneal administration of vehicle, bufalin (1 mg/kg), Erastin (20 mg/kg, a canonical ferroptosis inducer), or bufalin plus Erastin for 30 days. (**H**) Representative images of xenograft tumors harvested at the end of the experiment. (**I**) Body weight changes in mice during the treatment period. (**J**,**K**) Quantification of tumor weight (**J**) and tumor volume (**K**) in each group (*n* = 5). All data are presented as mean ± SD (* *p* < 0.05; ** *p* < 0.01; *** *p* < 0.001; **** *p* < 0.0001).

**Figure 8:**
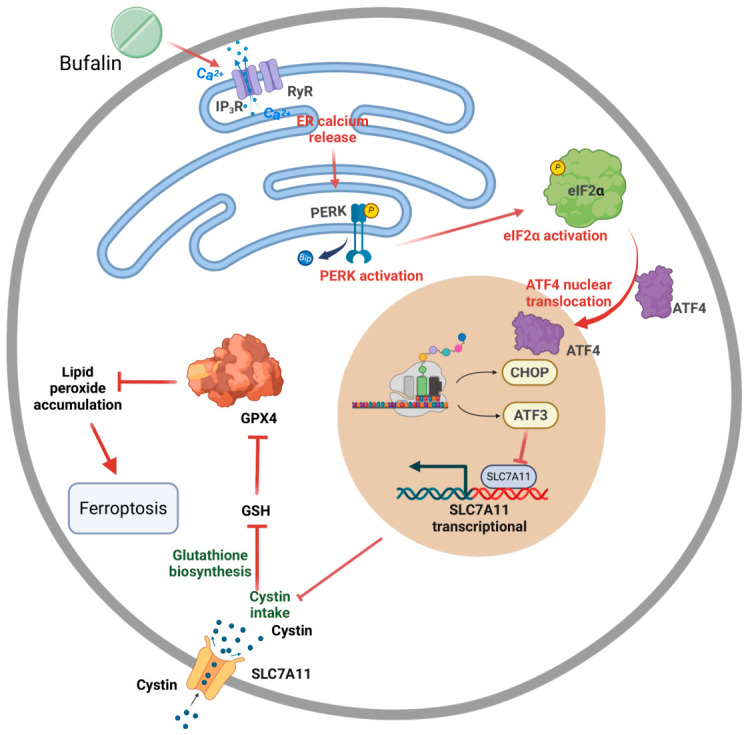
Graphical illustration of the mechanism underlying bufalin-induced ferroptosis in PDAC.

To further evaluate the contribution of ferroptosis to bufalin-mediated tumor suppression, PDAC cells were co-treated with bufalin and Fer-1. Fer-1 reduced bufalin-induced ROS, lipid peroxidation (Figure 7A,B and Figure S6A–D), and MDA accumulation and restored the GSH/GSSG ratio (Figure 7C,D). Fer-1 also partially attenuated the bufalin-induced changes in TFRC, GPX4, and SLC7A11 expression (Figure 7E) and rescued colony formation (Figure 7F). To extend these observations *in vivo*, we used a subcutaneous pancreatic tumor model and compared vehicle, bufalin, erastin, and combination treatment. Both bufalin and erastin inhibited tumor growth, and the combination produced a stronger suppressive effect than either monotherapy (Figure 7G–K). These findings provide evidence that bufalin promoted ER stress and ferroptosis in PDAC (Figure 8).

## 3. Discussion

Ferroptosis is increasingly recognized as a promising therapeutic vulnerability in PDAC, a malignancy characterized by profound treatment resistance and poor prognosis. Unlike apoptosis, which is often impaired in PDAC, ferroptosis is driven by iron-dependent lipid peroxidation and may therefore bypass conventional resistance mechanisms [38]. Natural products are an important source of ferroptosis-modulating compounds [44,45,46,47,48,49,50,51,52,53,54,55,56,57,58,59,60]. Although bufalin has long been known to exert antitumor effects [14,15,16], its role in ferroptosis regulation in PDAC has remained unclear. In the present study, our *in vitro* and in vivo data indicate that ferroptosis-related processes contribute importantly to bufalin-mediated antitumor activity in PDAC, although apoptosis- and pyroptosis-related pathways may also participate under our experimental conditions.

Integrated RNA-seq, DIA proteomic, and metabolomic analyses revealed coordinated alterations in Ca^2+^ homeostasis, ER stress/UPR signaling, oxidative stress defense, iron-related redox regulation, and lipid metabolic remodeling after bufalin treatment. Although transcriptomic and proteomic changes showed only moderate concordance at the individual molecule level, they converged on stress-response and metabolism-related pathways. These pathway signatures provided a basis for subsequent functional analyses of ER Ca^2+^ release, PERK/eIF2α/ATF4 activation, ATF3/SLC7A11 regulation, and ferroptosis-related biochemical changes. Thus, the multi-omics data helped define the biological context in which bufalin-induced ER stress and ferroptosis-related responses occur, rather than serving as independent proof of a linear causal pathway.

Ca^2+^ dyshomeostasis appears to be closely associated with the ER stress response induced by bufalin. Calcium-free medium experiments suggested that bufalin-induced cytosolic Ca^2+^ elevation was mainly derived from intracellular stores rather than extracellular influx. Pharmacological inhibition further showed that 2-APB attenuated cytosolic Ca^2+^ elevation and partially restored ER Ca^2+^ levels, whereas DAN showed weaker and cell line-dependent effects. Together with docking and SPR assays showing *in vitro* binding of bufalin to IP_3_R1/IP_3_R3, these results support the possibility that IP_3_R-associated ER Ca^2+^ release participates in the cellular response to bufalin. However, SPR provides *in vitro* binding evidence and does not by itself establish cellular target engagement or functional dependence on IP_3_R in intact PDAC cells. Moreover, the current cellular evidence for IP_3_R involvement is mainly based on pharmacological inhibition and Ca^2+^ imaging. Therefore, future studies using IP_3_R1/IP_3_R3 knockdown, knockout, isoform-specific rescue, or cellular target-engagement assays will be required to define the precise functional contribution of IP_3_R to bufalin-induced ER Ca^2+^ release and downstream stress responses.

ER stress was further linked to ferroptosis-related biochemical changes. Bufalin increased p-PERK/PERK and p-eIF2α/eIF2α ratios and upregulated ATF4, CHOP, and ATF3, consistent with activation of the PERK-associated ER stress pathway. Inhibition of ER stress by 4-PBA attenuated ROS accumulation, lipid peroxidation, GSH/GSSG imbalance, MDA production, and SLC7A11/GPX4 downregulation. These findings suggest that ER stress contributes to bufalin-induced redox imbalance and ferroptosis-related phenotypes in PDAC cells.

ATF3 may function as an important downstream mediator connecting ER stress to ferroptosis-related regulation. Bufalin increased ATF3 expression in parallel with PERK pathway activation, and modulation of ATF3 altered SLC7A11/GPX4 expression, oxidative stress, lipid peroxidation, and clonogenic growth. This interpretation is consistent with previous studies showing that ATF3 can repress SLC7A11 transcription and enhance ferroptotic sensitivity [61,62]. In this study, ChIP-qPCR showed enrichment of ATF3 at the predicted SLC7A11 promoter region. In addition, dual-luciferase promoter-reporter assays showed that ATF3 overexpression suppressed SLC7A11 promoter activity, and mutation of the predicted ATF3-binding motif largely attenuated this suppressive effect. These data provide functional promoter-level evidence supporting ATF3-mediated repression of SLC7A11.

Our findings also raise the possibility of a reciprocal reinforcement between ferroptosis and ER stress. In the present study, ER stress inhibition attenuated several ferroptosis-related phenotypes, suggesting that ER stress contributes to bufalin-induced lipid peroxidation and redox imbalance. This is consistent with recent evidence in pancreatic cancer showing that cannabigerol induces ER stress-mediated apoptosis and ferroptosis through the IRE1α-XBP1 axis [63], and that melatonin promotes ferroptosis through ATF4-dependent ER stress [64]. Conversely, ferroptosis-associated lipid peroxidation may further aggravate ER dysfunction, as ferroptotic agents can induce ER stress responses [65], and the ER membrane has been identified as an early site of lipid peroxidation during ferroptosis [66]. Thus, bidirectional interaction between ER stress and ferroptosis is biologically plausible, although it was not directly examined in the present study.

Several limitations should be noted. First, although docking and SPR support a physical interaction between bufalin and IP_3_R1/IP_3_R3 in vitro, stronger cellular evidence remains needed. SPR does not directly demonstrate cellular target engagement, and the current functional evidence for IP_3_R involvement remains indirect. Future studies using IP_3_R1/IP_3_R3 silencing, knockout, isoform-specific rescue, or cellular target-engagement approaches would help define whether IP_3_R is a direct and functionally indispensable upstream mediator of bufalin-induced Ca^2+^ dyshomeostasis. Second, although ChIP-qPCR and dual-luciferase reporter assays support ATF3 binding to and functional repression of the SLC7A11 promoter region, reporter assays based on promoter fragments do not fully reproduce the endogenous chromatin environment. Endogenous promoter editing or additional rescue experiments would further strengthen the conclusion that ATF3 directly regulates SLC7A11 transcription in this context. Third, several mechanistic inferences rely on pharmacological inhibitors, including 2-APB, 4-PBA, and Fer-1, all of which may have off-target or context-dependent effects. Therefore, inhibitor-based findings should be interpreted as supportive rather than definitive evidence for the involvement of Ca^2+^ release, ER stress, and ferroptosis-related lipid peroxidation in the response to bufalin. Fourth, although the multi-omics analyses revealed coordinated changes in Ca^2+^ homeostasis, ER stress/UPR signaling, oxidative stress defense, and ferroptosis-related metabolic programs, these findings remain primarily pathway-level and hypothesis-supporting. Because the overlap of differentially altered molecules across omics layers was limited and the datasets were generated from endpoint profiling, the multi-omics results cannot, by themselves, establish a linear causal cascade or define direct relationships among specific molecular components of the proposed model, such as IP_3_R, PERK, ATF3, SLC7A11, and GPX4.

In summary, this study shows that bufalin suppresses PDAC progression and is associated with Ca^2+^ dyshomeostasis, ER stress activation, and ferroptosis-related responses. The data suggest that IP_3_R-associated ER Ca^2+^ release, PERK/ATF3 signaling, SLC7A11/GPX4 suppression, and impaired antioxidant defense may jointly contribute to the antitumor effects of bufalin. More broadly, these findings highlight ER stress-ferroptosis crosstalk as a potential therapeutic vulnerability in PDAC, while indicating that further genetic and mechanistic validation is required to define the proposed signaling axis more rigorously.

## 4. Materials and Methods

### 4.1. Cell Lines and Cell Culture

Human PDAC cell lines MIA PaCa-2, PANC-1, BxPC-3, and AsPC-1 were obtained from the American Type Culture Collection (ATCC, Manassas, VA, USA). The murine Panc02 cell line was provided by the National Infrastructure of Cell Line Resource (Beijing, China). PANC-1 and Panc02 cells were cultured in Dulbecco’s modified Eagle’s medium (DMEM; Gibco, Waltham, MA, USA) supplemented with 10% fetal bovine serum (Gibco, Waltham, MA, USA), 100 U/mL penicillin, and 100 μg/mL streptomycin (Gibco, Waltham, MA, USA). MIA PaCa-2 cells were maintained in the same basal medium with the addition of 2.5% horse serum (Gibco, Waltham, MA, USA).

### 4.2. Reagents and Antibodies

The major chemical reagents used in this study are listed in Table 1, including their supplier information, catalog numbers, CAS numbers, molecular formulas, molecular weights, and purity.

The antibodies used for Western blotting are listed in Table 2, together with their sources, catalog numbers, and working dilutions.

### 4.3. Bioinformatics Analysis

mRNA expression profiles and corresponding clinical information were retrieved from UCSC Xena database (https://xenabrowser.net/datapages/, accessed on 13 January 2025), including the pancreatic cancer cohort from The Cancer Genome Atlas and datasets from the Genotype-Tissue Expression project. Ferroptosis-related genes were downloaded from FerrDb (http://www.zhounan.org/ferrdb, accessed on 20 January 2025), and ER stress-related gene sets were obtained from the Molecular Signatures Database (https://www.gsea-msigdb.org/gsea/index.jsp, accessed on 20 January 2025). Pathway activity was assessed using gene set variation analysis (GSVA). Samples were divided into high and low groups based on optimal cutoffs determined by the surv_cutpoint function from the survminer R package (version 4.3.1). Survival differences between groups were analyzed using Kaplan–Meier curves, with statistical significance evaluated by the log-rank test. Weighted gene co-expression network analysis was performed to identify gene modules that showed the strongest correlation with ferroptosis and ER stress scores. Functional enrichment analysis of genes within these modules was conducted using the clusterProfiler package, focusing on Gene Ontology (GO) terms and Kyoto Encyclopedia of Genes and Genomes (KEGG) pathways. A false discovery rate (FDR) of less than 0.05 was considered statistically significant.

### 4.4. High-Throughput RNA Sequencing and Gene Set Enrichment Analysis

RNA samples were isolated from MIA PaCa-2 cells treated with either 0 or 20 nM for 48 h bufalin (*n* = 3 per group) using TRIzol reagent (Sigma-Aldrich, St. Louis, MO, USA). RNA integrity and concentration were verified, with only samples exhibiting an RNA Integrity Number above 7 proceeding to sequencing. The Illumina platform (Illumina, San Diego, CA, USA) was used to perform RNA-seq experiments, and DESeq2 version 1.44.0 served as the analytical tool for conducting differential gene expression analysis. Gene set enrichment analysis (GSEA) was performed using the clusterProfiler package v4.12.0 with 1000 permutations of the gene sets. Enriched pathways or gene sets were considered statistically significant based on the criteria: normalized enrichment score (|NES|) > 1, adjusted *p* value < 0.05, and false discovery rate q < 0.05).

### 4.5. Proteomics Analysis of Bufalin-Treated PDAC Cells

PDAC cells treated with bufalin for 48 h were subjected to proteomic analysis data-independent acquisition mass spectrometry. For sample preparation, SDC buffer (5% SDC, 100 mM Tris-HCl, pH 8.5) was added directly to cells, followed by sonication and boiling for 15 min. Lysate samples were subjected to centrifugation at 14,000× *g* for 40 min, and the resulting supernatants were quantitated for protein concentration using a bicinchoninic acid protein assay kit (Beyotime Biotechnology, Shanghai, China; Cat. No. P0010S). For each sample, 15 micrograms of total protein was combined with 5X SDS-PAGE loading buffer, heated to boiling for 5 min, and subsequently resolved via 4–20% SDS-PAGE electrophoresis at 180 volts for 45 min.

For digestion, DTT was added for disulfide bond reduction (37 °C, 1.5 h), followed by IAA alkylation (room temperature, dark, 30 min). Trypsin (1:50, wt/wt) was incubated with samples at 37 °C overnight; peptide samples underwent desalting via an MCX chromatography column and were concentrated through vacuum centrifugation. The dried samples were reconstituted in 0.1% formic acid prior to analysis. Data-independent acquisition (DIA) mass spectrometry was performed on a Vanquish Neo LC system interfaced with an Orbitrap™ Astral™ mass spectrometer. Raw mass spectrometry data were analyzed with the DIA-NN 1.8.1 software, with an FDR set at ≤1%. Bioinformatic analyses, including PCA and GO/KEGG annotation, were performed using corresponding software and R packages.

### 4.6. Metabolomics Analysis of Bufalin-Treated PDAC Cells and Hierarchical Clustering Analysis of Differential Metabolites

PDAC cells exposed to bufalin for 48 h were subjected to untargeted metabolomic profiling. Following removal of the culture medium, cells were gently washed with prewarmed phosphate-buffered saline (PBS) prior to extraction. Metabolites were extracted and proteins precipitated using 800 μL of ice-cold methanol/acetonitrile (1:1, *v*/*v*). After centrifugation at 14,000× *g* for 20 min at 4 °C, the clarified supernatant was carefully transferred and evaporated to dryness under vacuum. The residue was subsequently dissolved in 100 μL of acetonitrile/water (1:1, *v*/*v*) and centrifuged to remove insoluble material. The final supernatant was subjected to liquid chromatography–mass spectrometry analysis. For quality control, pooled QC samples were generated by mixing equal aliquots (10 μL) from each specimen. These QC samples were analyzed after every five experimental samples during the analytical sequence.

Chromatography was conducted on a Vanquish UHPLC system linked to an Orbitrap Exploris™ 480 instrument. Separation was achieved on an ACQUITY BEH Amide column (2.1 × 100 mm, 1.7 μm) using a binary solvent system composed of aqueous ammonium acetate/ammonium hydroxide (25 mM, solvent A) and acetonitrile (solvent B), with gradient elution applied for resolution.

Raw mass spectrometry data were first transformed into MzXML format via ProteoWizard (v. 3.0.25329). Subsequent data processing included peak detection and alignment via XCMS, followed by annotation using the CAMERA tool. Multivariate analyses, including principal component analysis (PCA) and orthogonal partial least squares-discriminant analysis, were performed to evaluate metabolic variation between groups, accompanied by univariate comparisons using Student’s *t*-test. Metabolites exhibiting a variable importance in projection (VIP) value greater than 1 together with a *p* value below 0.05 were considered differentially abundant. Prior to hierarchical clustering, both samples and differential metabolites were standardized by subtracting the group mean and dividing by the root mean square. A distance matrix was computed, and hierarchical clustering was performed using an agglomerative approach that iteratively merges the closest clusters.

### 4.7. Cell Viability Assay and Cell Death Pathway Assays

Cell viability was evaluated using the Cell Counting Kit-8 (CCK-8; Beyotime Shanghai, China; Cat. No. C0038) according to the manufacturer’s protocol. Approximately 3 × 10^3^ cells were plated into each well of 96-well plates and cultured overnight to allow attachment. Cells were then exposed to varying concentrations of bufalin for the indicated time periods. After drug exposure, 10 µL of CCK-8 reagent was introduced into each well containing 100 µL of medium and incubated at 37 °C for 2 h. The optical density at 450 nm was subsequently recorded using a BioTek microplate reader (BioTek Instruments, Winooski, VT, USA).

96-well plates were plated with MIA PaCa-2, PANC-1, BxPC-3, and AsPC-1 cells and incubated at 37 °C overnight. Afterward, the existing solution was exchanged for 100 μL working solution of bufalin (20 nM), apoptosis inhibitor Z-VAD-FMK (20 μM), pyroptosis inhibitor MCC950 (10 μM), ferroptosis inhibitor Fer-1 (2.5 μM), the mixture of Z-VAD-FMK and bufalin, MCC950 and bufalin, or Fer-1 and bufalin. MTT assay under 490 nm was used to evaluate the cell death pathway after 24 h incubation.

### 4.8. Apoptosis Assay

Apoptosis was evaluated using an Annexin V-PE/7-AAD detection kit (BD Biosciences, Franklin Lakes, NJ, USA; Cat. No. 559763). MIA PaCa-2 and PANC-1 cells (1 × 10^5^ per well) were plated in 6-well plates and exposed to bufalin at the indicated concentrations for 48 h. Both attached and suspended cells were subsequently collected, rinsed with ice-cold PBS, and processed for staining following the manufacturer’s protocol. Samples were analyzed on a Beckman Coulter CytoFLEX flow cytometer (Beckman Coulter, Brea, CA, USA).

### 4.9. Colony Formation Assay

For clonogenic analysis, 1 × 10^3^ cells were seeded into each well of 6-well plates and allowed to attach for 48 h. Cells were then exposed to bufalin at the indicated concentrations for 24 h, after which the medium was replaced with fresh drug-free medium. The cultures were maintained for an additional 14 days to allow colony development. During this culture period, the medium was refreshed every three days. After the incubation, colonies were fixed using a 4% paraformaldehyde solution for 30 min and then stained with 0.1% crystal violet solution for another 30 min. Following a rinse with PBS, the colonies were imaged and examined under a light microscope.

### 4.10. Wound-Healing Assay

MIA PaCa-2 and PANC-1 cells were plated in 6-well culture plates and cultured to reach full confluency. A sterile 1 mL pipette tip was used to scratch the confluent cell monolayers and create uniform wounds. The culture medium was then refreshed with fresh complete medium supplemented with bufalin at concentrations of 0, 10, and 20 nM, and the cells were incubated for 48 h. Wound closure efficiency was assessed by capturing images under a phase contrast microscope at 0, 24, and 48 h post-scratching. All scratch-wound migration assays were repeated in at least three independent experimental replicates to ensure reproducibility.

### 4.11. Animal Studies

All animal procedures were conducted in accordance with institutional ethical regulations and were approved by the Ethics Committee of Fudan University (Approval No. FUSCC-IACUC-S220210178). Six-week-old male C57BL/6 mice were purchased from Shanghai SLAC Laboratory Animal Co., Ltd. (Shanghai, China) Orthotopic pancreatic allograft model were established by injecting Panc02 cells (5 × 10^5^) into the pancreas.

The bufalin doses used *in vivo* (0.5 and 1 mg/kg) were selected based on previously published mouse tumor studies reporting antitumor activity of bufalin within a similar dose range, generally around 0.5–2 mg/kg, while avoiding the higher-dose range associated with increased toxicity [67,68]. Therefore, 0.5 mg/kg was chosen as a low dose and 1 mg/kg as a higher dose to assess dose-dependent antitumor efficacy with acceptable tolerability.

Animals were maintained under specific pathogen-free conditions and subsequently allocated into three groups: control, low-dose bufalin (0.5 mg/kg), and high-dose bufalin (1 mg/kg), with six mice per group. Tumor growth was evaluated through bioluminescence imaging performed on an IVIS Lumina K Series III platform (PerkinElmer, Waltham, MA, USA). Upon euthanasia, tumors were excised, weighed, measured, and processed for immunohistochemistry and histopathological analyses.

For subcutaneous xenograft models, male C57BL/6 mice aged four weeks were obtained from Shanghai Jihui Laboratory Animal Co., Ltd. (Shanghai, China) Panc02 cells (1 × 10^6^) were implanted subcutaneously into the right flank of all mice. The mice were randomly divided into 4 groups receiving intraperitoneal injections (100 μL, every other day) as follows: control group (vehicle), bufalin group (0.5 mg/kg), Erastin (20 mg/kg), or bufalin plus Erastin (0.5 mg/kg + 20 mg/kg) for 28 days.

### 4.12. Transmission Electron Microscopy (TEM)

Cells processed according to the aforementioned protocol were harvested and then subjected to fixation with 2.5% glutaraldehyde at 4 °C for a 2 h incubation period and processed for ultrastructural examination by Lianlan Biotechnology (Shanghai, China).

### 4.13. Measurements of Ca^2+^

For the detection of intracellular Ca^2+^ levels, cells were incubated with 1 μM Fluo-4 AM (Beyotime, Shanghai, China; Cat. No. S1061S) fluorescent dye, followed by washing steps. The intracellular Ca^2+^ concentration was determined by measuring the fluorescence intensity of Fluo-4 AM using a BioTek microplate reader (BioTek Instruments, Winooski, VT, USA).

To evaluate ER Ca^2+^ content, the BBcellProbe^®^ C93 staining reagent (Bestbio, Cat. BB-481159) was initially thawed in a 37 °C water bath and then diluted 1:1000 with Hank’s Balanced Salt Solution (HBSS) to prepare the working solution. Harvested target cells were washed three times with HBSS, after which the Fluo-4 AM working solution was applied. The cells were subsequently cultured at 37 °C for 2 h in a humidified atmosphere containing 5% CO_2_. Following three additional HBSS washes, the cells were resuspended in HBSS buffer and incubated again at 37 °C for 30 min. Fluorescence intensity was quantified on a BioTek microplate reader, with excitation and emission wavelengths set at 565 nm and 580 nm, respectively.

### 4.14. Molecular Docking and Visualization

Molecular docking experiments were conducted between bufalin and targets utilizing Autodock Vina software (Version 1.5.7; retrieved from https://vina.scripps.edu/, accessed on 20 November 2025). 2D molecular docking analyses were performed utilizing the PROTEINS PLUS web-based platform (accessible at https://proteins.plus/, accessed on 22 November 2025). Significant binding interactions between the target proteins and the small molecules were identified, with an affinity score threshold set at ≤−5.0 kcal/mol.

### 4.15. Immunohistochemistry

Tumor specimens were fixed in paraformaldehyde, embedded in paraffin, and subsequently sectioned. To eliminate endogenous peroxidase activity, sections were treated with 3% hydrogen peroxide. Antigen retrieval was carried out using either citrate buffer or a high-pH buffer (Solarbio, Beijing, China) through heat induction. Blockade of non-specific antibody binding was achieved using goat serum (Sangon Biotech, Shanghai, China) as the blocking agent for the tissue sections. The sections were then subjected to overnight incubation at 4 °C with primary antibodies that specifically target the Ki67 (Abcam, ab15580, 1:2000), p-PERK (Abconal Biotech, Wuhan, China; AP1501, 1:200), PERK (Abconal Biotech, Wuhan, China; A18196, 1:200), EIF2A (Abconal Biotech, Wuhan, China; A2221, 1:200), p-EIF2A (Abconal Biotech, Wuhan, China; AF3087, 1:200), CHOP (Beyotime Biotechnology, Shanghai, China;AF6684, 1:200), ATF3 (Abcam, Waltham, MA, USA; ab191513, 1:200), GPX4 (Abcam, ab125066, 1:200), and SLC7A11 (Proteintech, Rosemont, IL, USA; 26864-1-ap, 1:200).

### 4.16. Western Blot Analysis

Total protein lysates were prepared in RIPA buffer supplemented with protease and phosphatase inhibitor cocktails (NCM Suzhou, China; P002). After denaturation in Laemmli sample buffer, proteins were separated by 10% SDS-PAGE and transferred onto polyvinylidene fluoride membranes. Membranes were blocked in 5% skim milk prepared in TBST and incubated overnight at 4 °C with specific primary antibodies. After washing, HRP-linked secondary antibodies were applied for 2 h at room temperature. Immunoreactive signals were detected using enhanced chemiluminescence reagents and quantified by densitometric analysis with ImageJ (version 1.0; National Institutes of Health, Bethesda, MD, USA).

### 4.17. Detection of ROS and Lipid Peroxidation

MIA PaCa-2, PANC-1, and BxPC-3 cells were exposed to bufalin for 48 h, followed by washing with PBS. To assess intracellular ROS, cells were stained with 5 mM DCFH-DA (S0033M, Beyotime Biotechnology, Shanghai, China). For the detection of lipid peroxidation, 5 µM BODIPY 581/591 C11 (S0043S, Beyotime Biotechnology, Shanghai, China) was used. Fluorescence intensity was subsequently measured and quantified using flow cytometry (Beckman Coulter, Brea, CA, USA).

### 4.18. Malondialdehyde (MDA) Assay, GSH and GSSG Assay

Following 48 h of bufalin treatment, cells were lysed via sonication, and concentrations of total protein were quantified via BCA assay. MDA levels, indicative of lipid peroxides, were measured with a lipid peroxides assay kit (Beyotime Biotechnology, Shanghai, China) and normalized to total protein content. Additionally, the ratio of GSH to oxidized glutathione disulfide (GSSG) was determined via a dedicated assay kit (Beyotime Biotechnology, Shanghai, China), with all procedures performed in adherence to the manufacturer’s recommended protocol.

### 4.19. Plasmid Construction and Lentivirus Production

shRNAs targeting ATF3 were cloned into pLKO.1-puro vectors (TSINGKE, Beijing, China). The sequences were: GCTGAACTGAAGGCTCAGATT and CTTCATCGGCCCACGTGTATT. ATF3 overexpression constructs were cloned into pLVX-puro vectors (TSINGKE, Beijing, China). Co-transfection of HEK-293T cells with lentiviral expression plasmids and the packaging plasmids pMD2.G and psPAX2 (at a 4:3:1 ratio) was performed to generate lentiviral particles, with transfections carried out using a lipid-based transfection reagent for optimal efficiency. Control groups included cells transduced with scrambled shRNA or empty vector constructs (TSINGKE, Beijing, China).

### 4.20. Immunofluorescence Co-Expression Analysis

MIA PaCa-2 and PANC-1 cell lines with stable overexpression or knockdown of ATF3 were seeded into confocal imaging dishes at a density of 1 × 10^5^ cells per well and cultured for 48 h. Cells were fixed in 4% paraformaldehyde for 30 min, followed by permeabilization with 1% Triton X-100 for 10 min. After blocking in 5% bovine serum albumin (BSA) for 40 min, samples were incubated with the appropriate primary antibodies. To examine the subcellular distribution of specific target proteins, cell samples were incubated overnight at 4 °C with primary antibodies against SLC7A11 (Abcam, Waltham, MA, USA; ab307601, diluted 1:100) and ATF3 (Abcam, Waltham, MA, USA; ab191513, diluted 1:100), followed three 5 min washes with PBS. Alexa Fluor 488-conjugated anti-rabbit IgG or Alexa Fluor 594-conjugated anti-mouse IgG secondary antibodies (Proteintech, Rosemont, IL, USA; 1:500 dilution) were applied for 1 h at room temperature under light-protected conditions. Nuclei were counterstained with DAPI (1 μg/mL in PBS) for 5 min at room temperature, followed by two final PBS washes. Confocal images were acquired using a laser scanning confocal microscope (Olympus, Tokyo, Japan). For quantitative assessment of co-expression, ImageJ software ImageJ (version 1.0; National Institutes of Health, Bethesda, MD, USA) was employed to determine the mean fluorescence intensity of SLC7A11 and ATF3 in individual cells.

### 4.21. Surface Plasmon Resonance (SPR) Assay

Surface plasmon resonance (SPR) assays were performed using a CM5 sensor chip to evaluate the binding affinity between bufalin and IP_3_R proteins. Protein immobilization was conducted by the standard amine-coupling method. Briefly, the running buffer and interaction buffer consisted of 1 × PBS-P+ (pH 7.4), and the interaction buffer additionally contained 5% (*v*/*v*) DMSO. The sensor chip was activated with a mixture of 1-ethyl-3-(3-dimethylaminopropyl) carbodiimide and N-hydroxysuccinimide at a flow rate of 10 μL/min for 420 s. Recombinant ligand proteins were diluted to 50 μg/mL in sodium acetate buffer (pH 4.5) and immobilized on the flow cell at a flow rate of 10 μL/min for 420 s. Residual active groups were then blocked with ethanolamine at 10 μL/min for 420 s. A reference flow cell was prepared using protein-free acetate buffer under the same coupling conditions.

For binding analysis, bufalin was first diluted to 16 μM in interaction buffer and then serially diluted to 0.125 μM. Different concentrations of bufalin were injected over the immobilized protein surface at a flow rate of 30 μL/min. The association and dissociation phases were monitored for 60 s and 90 s, respectively. After each cycle, the sensor surface was regenerated with 10 mM glycine-HCl (pH 2.0) for 5 min. All data were analyzed using the instrument’s standard evaluation software.

### 4.22. Chromatin Immunoprecipitation (ChIP) Assay and Luciferase Reporter Assay

ChIP assays were performed in MIA PaCa-2 and PANC-1 cells to verify the binding of ATF3 to the SLC7A11 promoter. Cells were cross-linked with 1% formaldehyde, quenched with 0.125 M glycine, and lysed for chromatin extraction. The chromatin was sonicated to generate DNA fragments of approximately 300–1000 bp, pre-cleared with Protein A/G magnetic beads, and then incubated overnight at 4 °C with anti-ATF3 antibody or normal rabbit IgG. Immune complexes were captured with Protein A/G magnetic beads, washed sequentially, and eluted. After reversal of cross-links, DNA was purified and analyzed by ChIP-qPCR using primers targeting the predicted ATF3-binding regions within the SLC7A11 promoter. Input DNA served as an internal control, and IgG was used as a negative control.

For the luciferase reporter assay, the SLC7A11 promoter fragment containing the predicted ATF3-binding site was amplified from genomic DNA and inserted into the pGL3-Basic luciferase reporter vector. A mutant reporter construct carrying substitutions in the predicted ATF3-binding motif was generated by site-directed mutagenesis. HEK-293T cells were co-transfected with the wild-type or mutant SLC7A11 promoter-reporter plasmid, ATF3 overexpression plasmid or empty vector, and a Renilla luciferase plasmid as an internal control. After transfection, firefly and Renilla luciferase activities were measured using a dual-luciferase reporter assay system according to the manufacturer’s protocol. Relative luciferase activity was calculated by normalizing firefly luciferase activity to Renilla luciferase activity.

### 4.23. 4-HNE Immunofluorescence Staining

To evaluate lipid peroxidation in vivo, 4-HNE immunofluorescence staining was performed on PDAC xenograft tissues from control- and bufalin-treated mice. Tumor tissues were fixed in 4% paraformaldehyde, embedded in paraffin, and sectioned at 5 μm. Sections were deparaffinized, rehydrated, subjected to antigen retrieval in citrate buffer (pH 6.0), and blocked with 5% BSA. The sections were then incubated overnight at 4 °C with an anti-4-HNE primary antibody, followed by incubation with a fluorescent secondary antibody and nuclear counterstaining with DAPI.

### 4.24. Statistical Analysis

Statistical analyses were performed using GraphPad Prism (version 10.1.1; GraphPad Software Inc., San Diego, CA, USA). Data are presented as mean ± standard deviation (SD). Before inferential analysis, data distribution was assessed for normality using the Shapiro-Wilk test, and homogeneity of variances was evaluated using Brown-Forsythe test. For comparisons between two groups, a two-tailed unpaired Student’s *t*-test was used when data met the assumptions of normality and equal variance; otherwise, the Mann-Whitney U test was applied. For comparisons among more than two groups, one-way analysis of variance followed by Tukey’s post hoc test was used when the assumptions of normality and variance homogeneity were satisfied; otherwise, the Kruskal-Wallis test followed by Dunn’s multiple-comparisons test was performed. For *in vivo* experiments with relatively small sample sizes (*n* = 5–6 per group), assumption checks for normality and variance homogeneity were performed before selecting the appropriate statistical test. Overall survival was estimated using the Kaplan-Meier method, and differences between groups were analyzed using the log-rank test. A *p* value < 0.05 was considered statistically significant.

## 5. Conclusions

In conclusion, this study shows that bufalin exerts antitumor effects against PDAC both *in vitro* and *in vivo*. Integrative transcriptomic, proteomic, metabolomic, and bioinformatic analyses revealed coordinated alterations in calcium homeostasis, ER stress/UPR signaling, oxidative stress defense, and ferroptosis-related metabolic pathways after bufalin treatment. Functional experiments further linked bufalin treatment to ER Ca^2+^ dysregulation, PERK/ATF3 activation, SLC7A11/GPX4 downregulation, glutathione depletion, and lipid peroxidation. Together, these findings suggest that bufalin suppresses PDAC progression through coordinated ER stress- and ferroptosis-related responses, highlighting ER stress-ferroptosis crosstalk as a potential therapeutic vulnerability in PDAC.

## Figures and Tables

**Table 1:** Reagents used in this study.

Regent Name	Molecular Formula	CAS No.	Molecular Weight	Purity	Cat. No.	Source
Bufalin	C_24_H_34_O_4_	465-21-4	386.52	99.86%	HY-N0877	MedChemExpress (Shanghai, China)
Dantrolene (DAN)	C_14_H_10_N_4_O_5_	7261-97-4	314.26	98.38%	HY-12542	MedChemExpress (Shanghai, China)
4-Phenylbutyric acid (4-PBA)	C_10_H_12_O_2_	1821-12-1	164.20	99.98%	HY-A0281	MedChemExpress (Shanghai, China)
2-Aminoethyl diphenylborinate (2-APB)	C_14_H_16_BNO	524-95-8	225.09	99.86%	HY-W009724	MedChemExpress (Shanghai, China)
Ferrostatin-1 (Fer-1)	C_15_H_22_N_2_O_2_	347174-05-4	262.35	99.71%	HY-100579	MedChemExpress (Shanghai, China)
Z-VAD-FMK	C_22_H_30_FN_3_O_7_	187389-52-2	467.49	98%	C1202	Beyotime Biotechnology (Shanghai, China)
MCC950	C_20_H_24_N_2_O_5_S	210826-40-7	404.4799	97%	T6887	TargetMol Chemicals (Wellesley Hills, MA, USA)
Erastin	C_30_H_31_ClN_4_O_4_	571203-78-6	547.04	99.75%	T1765	TargetMol Chemicals (USA)

**Table 2:** Antibodies used in this study.

Antibody	Company	Cat. No.	Dilution Ratio
ATF3	Abcam (Waltham, MA, USA)	ab254268	1:1000
ATF4	Cell Signaling Technology (Danvers, MA, USA)	11815	1:1000
p-PERK	Abconal Biotech (Wuhan, China)	AP1501	1:1000
PERK	Abconal Biotech Wuhan, China)	A18196	1:1000
phospho-eIF2α (Ser51)	Affinity Biosciences (Cincinnati, OH, USA)	AF3087	1:1000
eIF2α	Abconal Biotech Wuhan, China)	A2221	1:1000
CHOP	Beyotime Biotechnology (Shanghai, China)	AF6684	1:500
TFRC	Beyotime Biotechnology (Shanghai, China)	AG3381	1:500
GPX4	Beyotime Biotechnology (Shanghai, China)	AF7020	1:500
SLC7A11	Beyotime Biotechnology (Shanghai, China)	AF7992	1:500
GAPDH	Proteintech (Rosemont, IL, USA)	60004-1-Ig	1:10,000
β-Actin	Proteintech (Rosemont, IL, USA)	20536-1-AP	1:10,000
Secondary antibodies	Proteintech (Rosemont, IL, USA)	SA00001-1, SA00001-2	1:10,000

## Data Availability

The original contributions presented in this study are included in the article/Supplementary Material. Further inquiries can be directed to the corresponding author.

## Supplementary Materials

The following supporting information can be downloaded at: https://www.mdpi.com/article/10.3390/ijms27104373/s1.

## Abbreviations

2-APB	2-Aminoethyl diphenylborinate
4-PBA	4-Phenylbutyric acid
BCA	Bicinchoninic acid protein
ChIP	Chromatin immunoprecipitation
DAN	Dantrolene
DEGs	Differentially expressed genes
DEPs	Differentially expressed proteins
DIA	Data-independent acquisition
ER	Endoplasmic reticulum
FDR	False discovery rate
Fer-1	Ferrostatin-1
GPX4	Glutathione peroxidase 4
GO	Gene ontology
GSH	Glutathione
GSSG	Oxidized glutathione disulfide
GSVA	Gene set variation analysis
HBSS	Hank’s balanced salt solution
KEGG	Kyoto encyclopedia of genes and genomes
MDA	Malondialdehyde
PDAC	Pancreatic ductal adenocarcinoma
PBS	Phosphate-buffered saline
RyR	Ryanodine receptor
SD	Standard deviation
TEM	Transmission electron microscopy
UPR	Unfolded protein response
VIP	Variable importance in projection
SPR	Surface plasmon resonance
WGCNA	Weighted gene co-expression network analysis
