# Supplementary material for: Bufalin Suppresses Pancreatic Ductal Adenocarcinoma Through ER Stress-Ferroptosis Crosstalk Associated with IP3R-Linked Ca2+ Dysregulation and ATF3/SLC7A11 Regulation
Source: Int J Mol Sci. 2026 May 14;27(10):4373. doi: 10.3390/ijms27104373 (PMC13207934; doi:10.3390/ijms27104373)

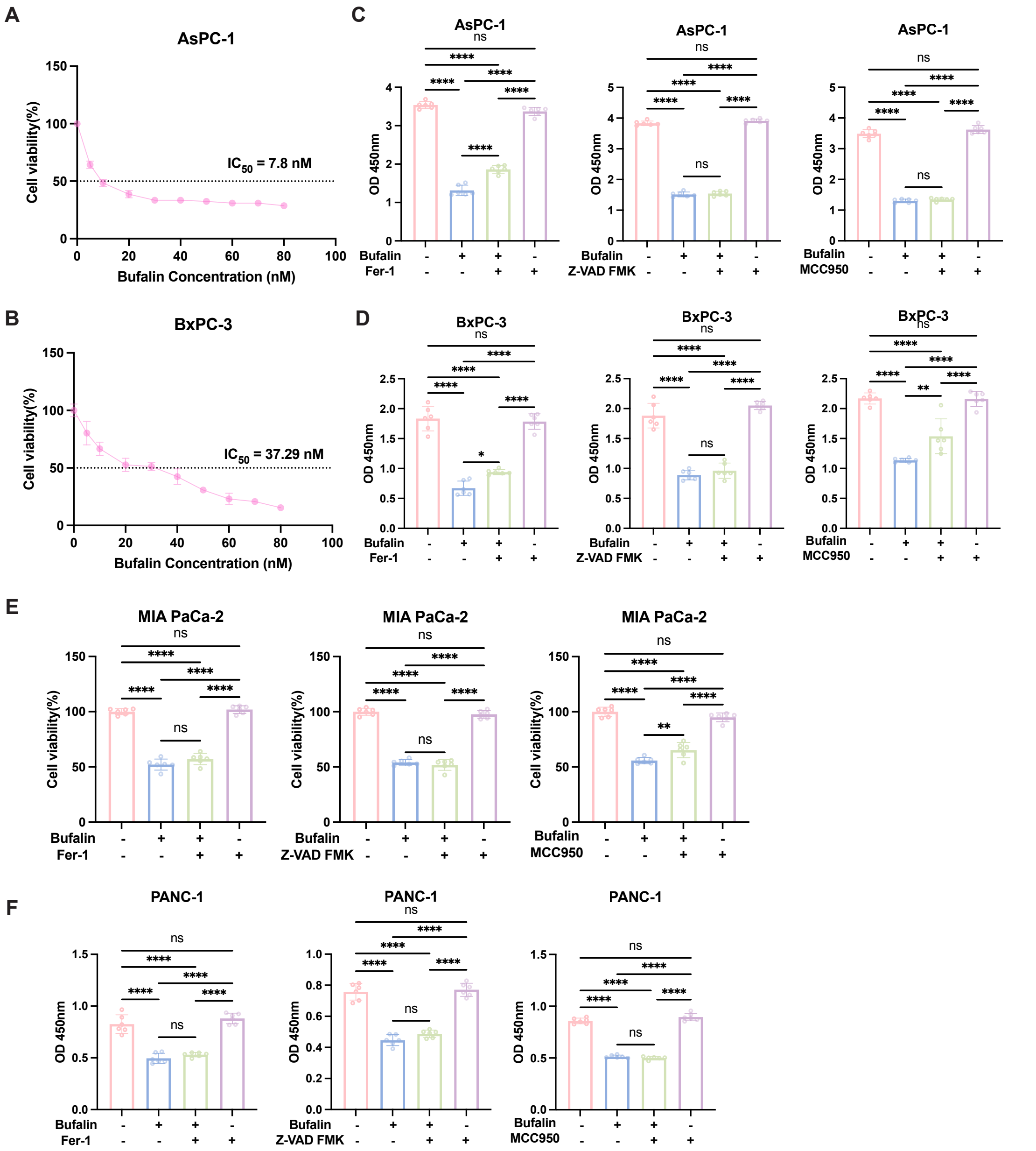

Figure S1. Bufalin induces ferroptosis-dependent cell death in pancreatic cancer cells.

(A, B) Dose-response curves of AsPC-1 (A) and BxPC-3 (B) cells treated with increasing concentrations of bufalin (0-80 nM) for 48 h. Cell viability was assessed by CCK-8 assay, and the half-maximal inhibitory concentration (IC_{50}) was calculated. (C–F) Cell viability of pancreatic cancer cell lines (AsPC-

1, BxPC-3, MIA PaCa-2, PANC-1) treated with bufalin (at their respective IC_{50} concentrations) alone or in combination with the ferroptosis inhibitor ferrostatin-1 (Fer-1, 1 μM), apoptosis inhibitor Z-VAD-FMK (20 μM), or necroptosis inhibitor MCC950 (10 μM) for 48 h. Cell viability was measured by CCK-8 assay (OD_{450} nm). Data are presented as mean ± SD. *$p < 0.05$, **$p < 0.01$, ***$p < 0.001$, ****$p < 0.0001$; ns, not significant (one-way ANOVA with Tukey's post hoc test).

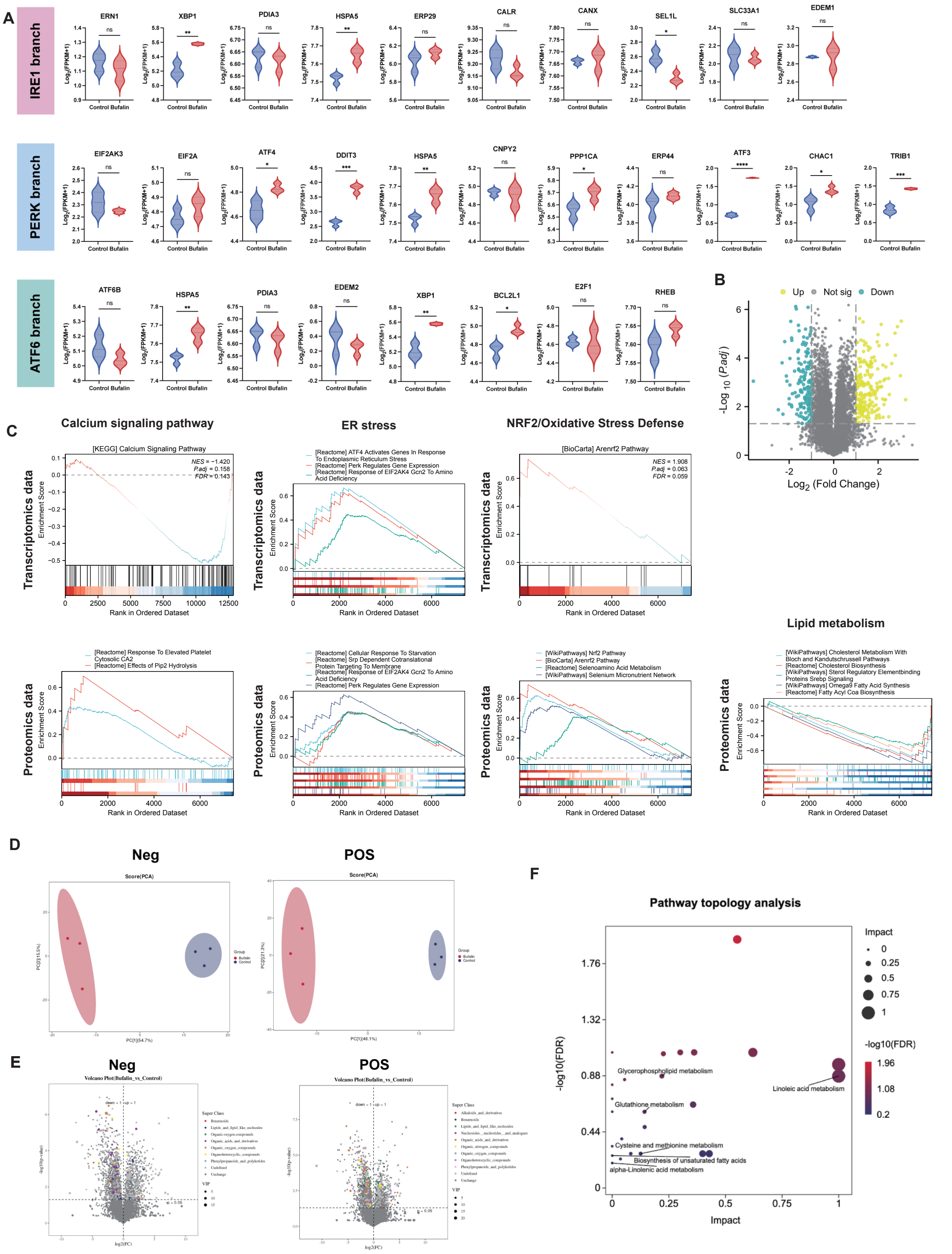

Figure S2. Multi-omics profiling of bufalin-induced molecular changes in PDAC cells.

(A) Expression levels of mRNAs encoding representative genes involved in the three canonical UPR branches, including IRE1, PERK, and ATF6 signaling, in control and bufalin-treated cells. (B) Volcano

plot of DIA proteomic data showing differentially expressed proteins between bufalin-treated and control cells. (C) Joint GSEA of RNA-seq and DIA proteomic datasets showing pathway-level alterations in calcium signaling, ER stress/UPR, NRF2/oxidative stress defense, and lipid metabolism. (D) PCA plots of untargeted metabolomic profiling data under negative ion mode (NEG) and positive ion mode (POS). (E) Volcano plots of differential metabolites identified by untargeted metabolomics under NEG and POS modes. (F) Pathway topology analysis showing the functional relevance of KEGG-enriched metabolic pathways.

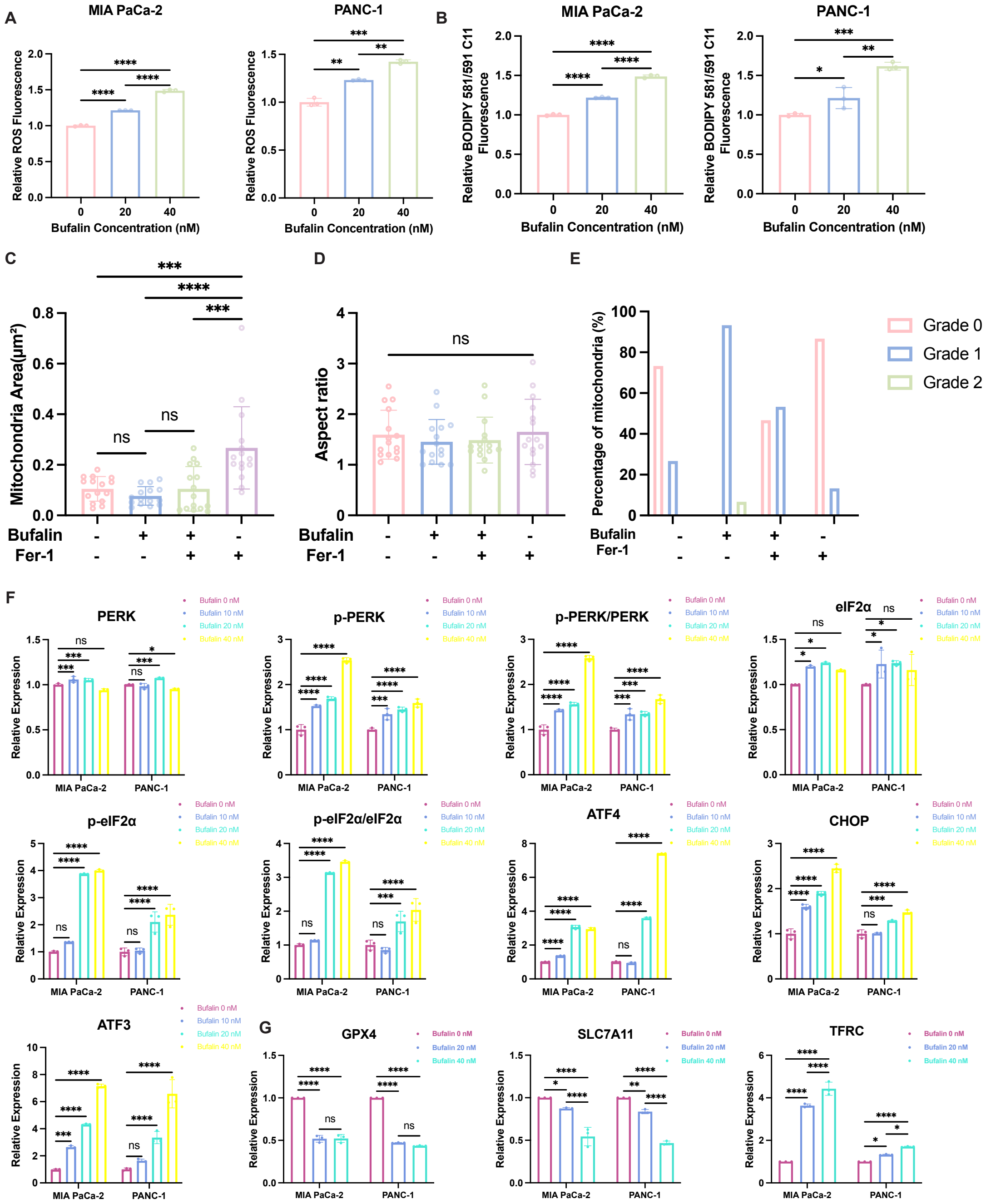

Figure S3. Bufalin induces oxidative stress, mitochondrial morphological changes, and PERK/eIF2α/ATF4/CHOP signaling activation in pancreatic cancer cells.

(A, B) Intracellular ROS levels (A) and lipid peroxidation levels (B) in MIA PaCa-2 and PANC-1 cells

treated with increasing concentrations of bufalin (0, 20, 40 nM) for 48 h, detected by DCFH-DA and BODIPY 581/591 C11 staining, respectively. (C-E) Mitochondrial morphology analysis in MIA PaCa-2 cells treated with bufalin (20 nM) with or without Fer-1 (1 μM) for 48 h. (C) Quantification of mitochondrial area. (D) Quantification of mitochondrial aspect ratio. (E) Distribution of mitochondrial morphological grades (Grade 0: normal; Grade 1: intermediate; Grade 2: fragmented). (F, G) Relative protein expression levels of PERK pathway-related proteins (PERK, p-PERK, p-eIF2α, eIF2α, ATF4, CHOP, ATF3) (F) and ferroptosis-related proteins (GPX4, SLC7A11, TFRC) (G) in MIA PaCa-2 and PANC-1 cells treated with bufalin (0, 10, 20, 40 nM) for 48 h. Data are presented as mean ± SD. *$p < 0.05$, **$p < 0.01$, ***$p < 0.001$, ****$p < 0.0001$; ns, not significant (one-way ANOVA with Tukey's post hoc test or two-way ANOVA, as appropriate).

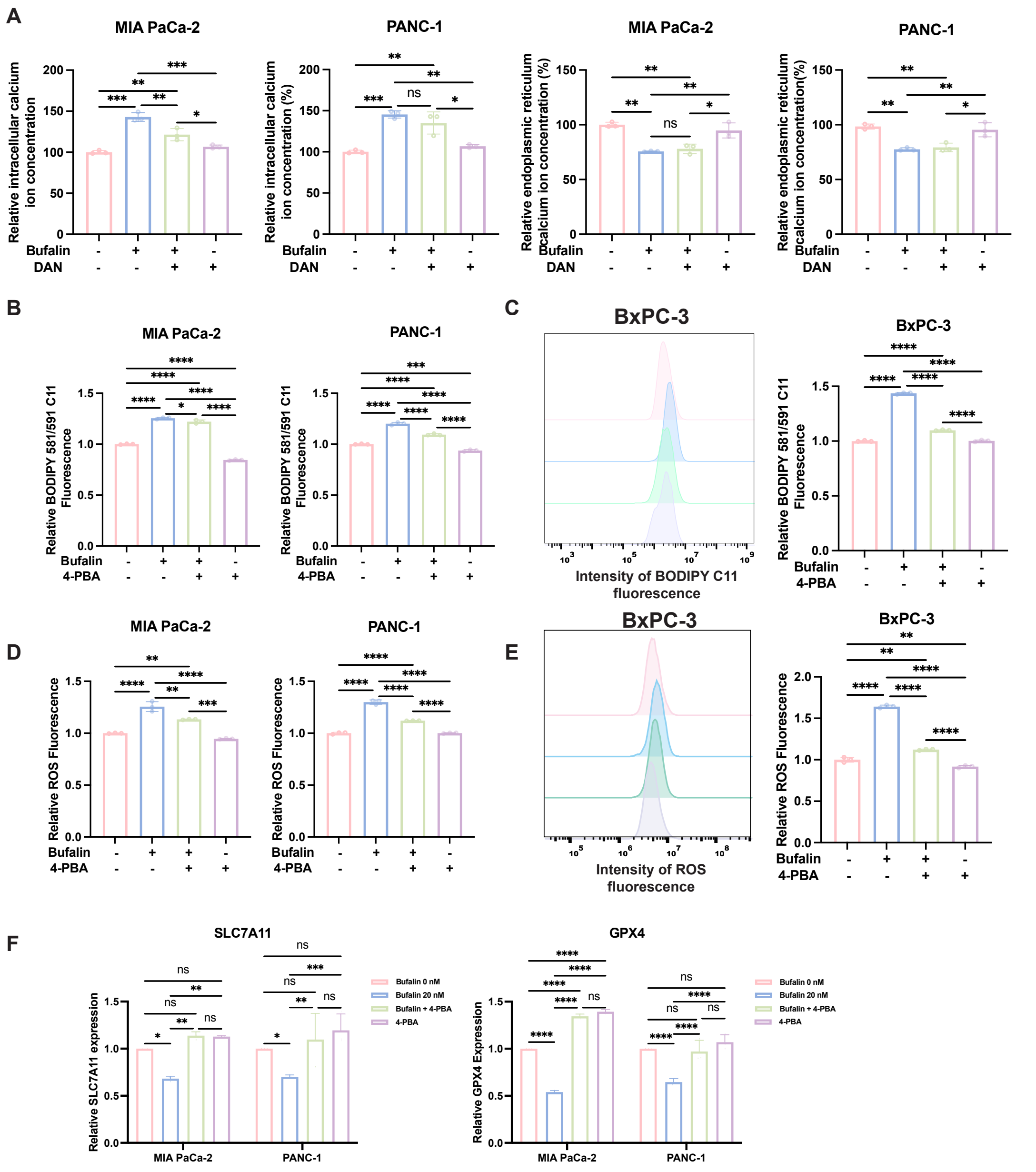

Figure S4. Bufalin promotes ER calcium release and ER stress to induce ferroptosis in pancreatic cancer cells.

(A) Relative intracellular calcium ion concentration and ER calcium ion concentration in MIA PaCa-2 and PANC-1 cells treated with bufalin (20 nM) alone or in combination with the ER calcium release inhibitor Dantrolene (DAN) for 24 h. (B, C) Relative lipid peroxidation levels (BODIPY 581/591 C11 fluorescence) in MIA PaCa-2, PANC-1 and BxPC-3 cells treated with bufalin alone or combined with

4-PBA. (D, E) Relative intracellular ROS levels in MIA PaCa-2, PANC-1 and BxPC-3 cells treated with bufalin alone or combined with 4-PBA. (F) Relative protein expression levels of ferroptosis-related proteins SLC7A11 and GPX4 in MIA PaCa-2 and PANC-1 cells treated with bufalin (0, 20 nM) alone or in combination with 4-PBA for 48 h. Data are presented as mean ± SD. *p < 0.05, **p < 0.01, ***p < 0.001, ****p < 0.0001; ns, not significant (one-way ANOVA with Tukey's post hoc test).

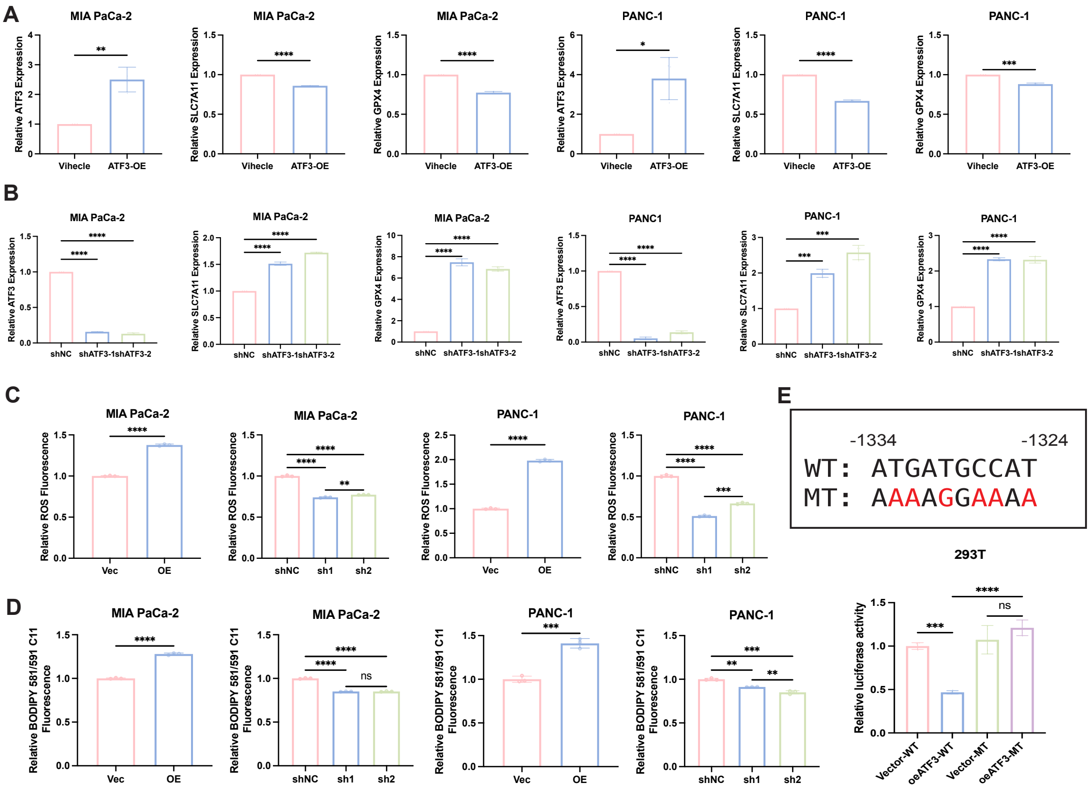

Figure S5. ATF3 regulates the SLC7A11/GPX4 axis, redox homeostasis, lipid peroxidation, and SLC7A11 promoter activity.

(A) Relative protein expression levels of ATF3, SLC7A11, and GPX4 in MIA PaCa-2 and PANC-1 cells transfected with ATF3 overexpression vector (ATF3-OE) or empty vector. (B) Relative protein expression levels of ATF3, SLC7A11, and GPX4 in MIA PaCa-2 and PANC-1 cells transfected with

ATF3 shRNA (shATF3-1 or shATF3-2) or non-targeting control shRNA (shNC). (C) Intracellular ROS levels in MIA PaCa-2 and PANC-1 cells with ATF3 overexpression or knockdown, measured by flow cytometry. (D) Lipid peroxidation levels in MIA PaCa-2 and PANC-1 cells with ATF3 overexpression or knockdown, detected using BODIPY 581/591 C11 fluorescence. (E) Dual-luciferase reporter assay showing the effect of ATF3 overexpression on wild-type (WT) or mutant (MT) SLC7A11 promoter activity in 293T cells. The predicted ATF3-binding motif in the SLC7A11 promoter was mutated from ATGATGCCAT to AAAAGGAAAA. Data are presented as mean ± SD. ns, not significant; *$p < 0.05$, **$p < 0.01$, ***$p < 0.001$, ****$p < 0.0001$.

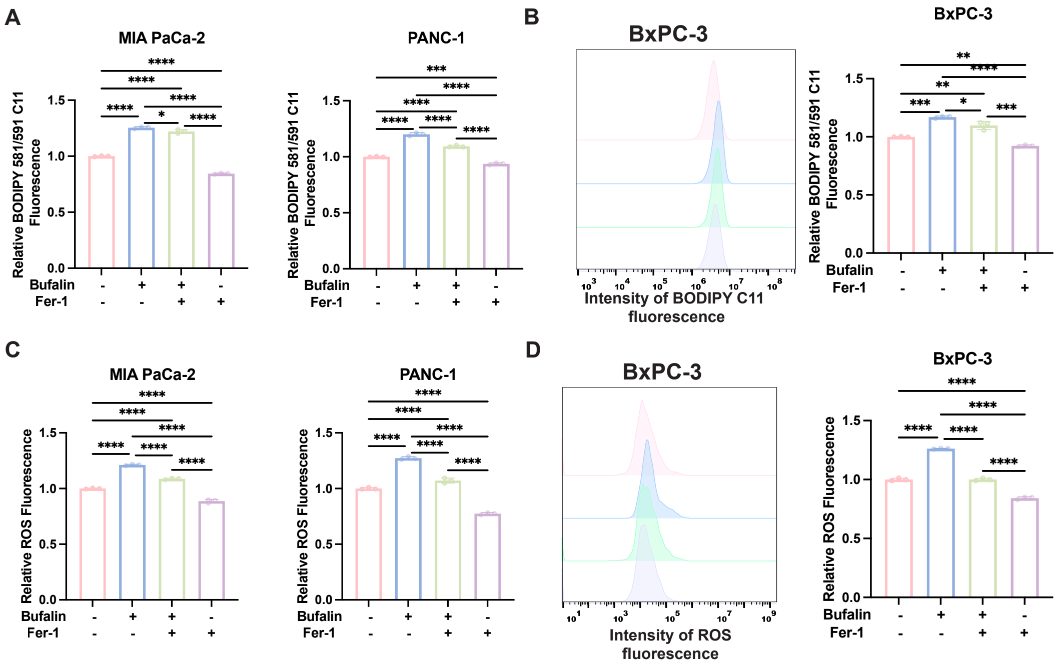

Figure S6. Bufalin induces lipid peroxidation and ROS accumulation via ferroptosis in pancreatic cancer cells.

(A, B) Lipid peroxidation levels in pancreatic cancer cells treated with bufalin alone or combined with the ferroptosis inhibitor ferrostatin-1 (Fer-1). Representative flow cytometry histograms and quantitative analysis of BODIPY 581/591 C11 fluorescence in MIA PaCa-2, PANC-1 (A) and BxPC-3 (B) cells are

shown. (C, D) Intracellular ROS levels in pancreatic cancer cells treated with bufalin alone or combined with Fer-1. Representative flow cytometry histograms and quantitative analysis of DCFH-DA fluorescence in MIA PaCa-2, PANC-1 (C) and BxPC-3 (D) cells are shown. All data are presented as mean ± SD. **p* < 0.05, ***p* < 0.01, ****p* < 0.001, *****p* < 0.0001 (one-way ANOVA with Tukey's post hoc test).

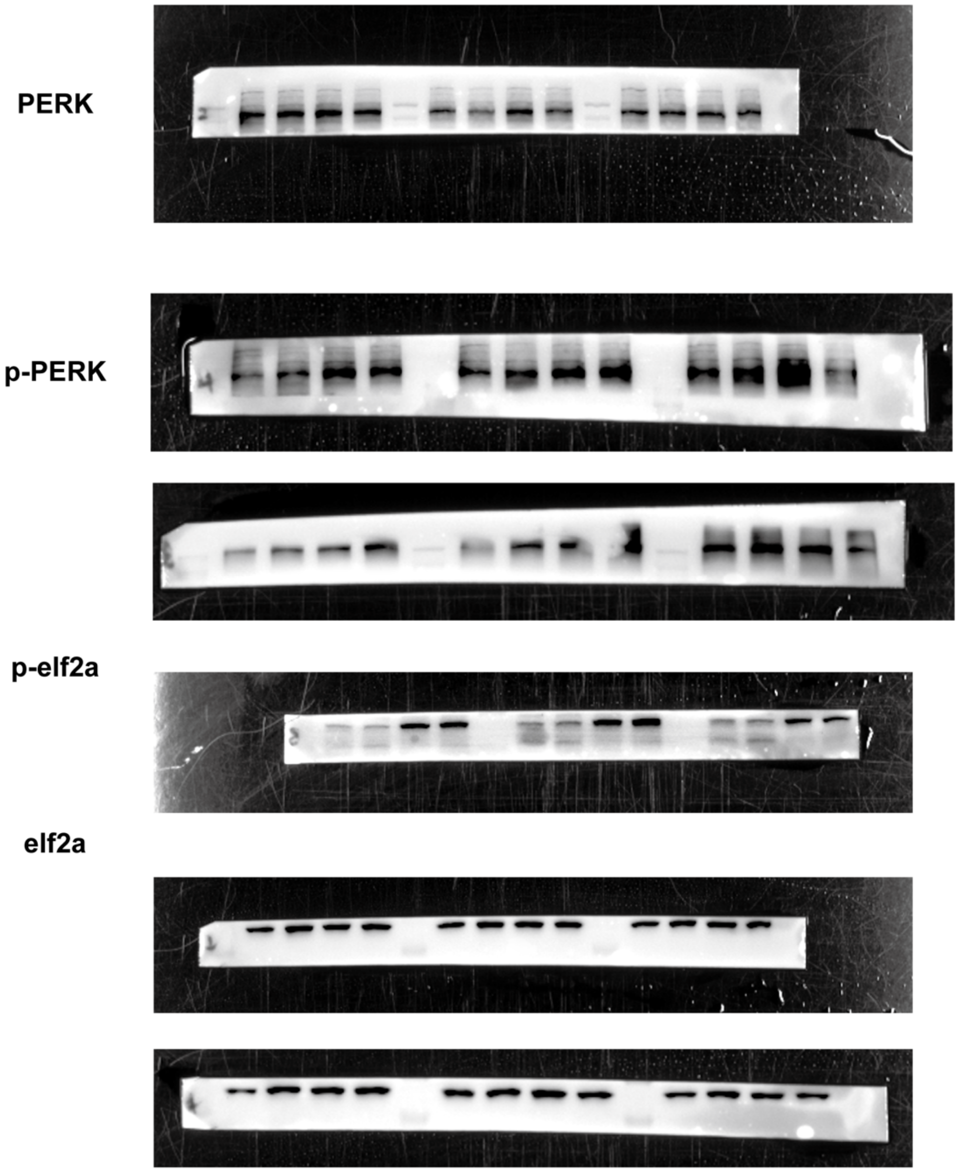

ATF4

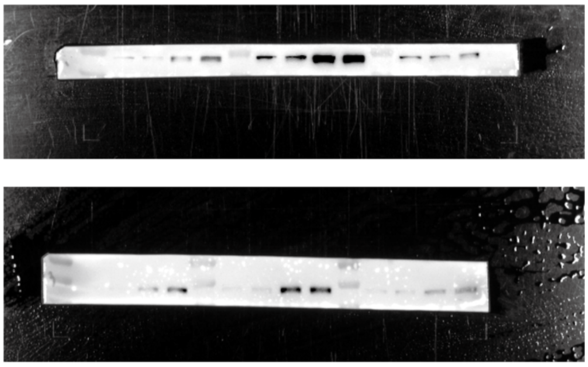

CHOP

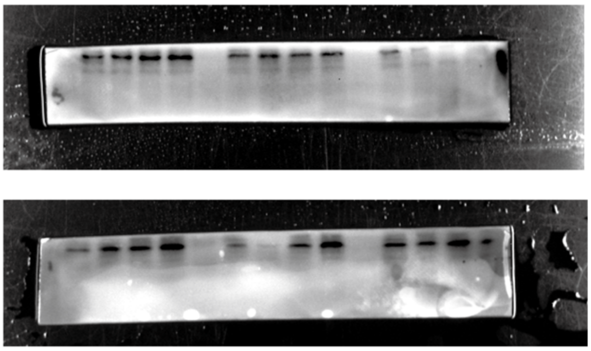

TFRC

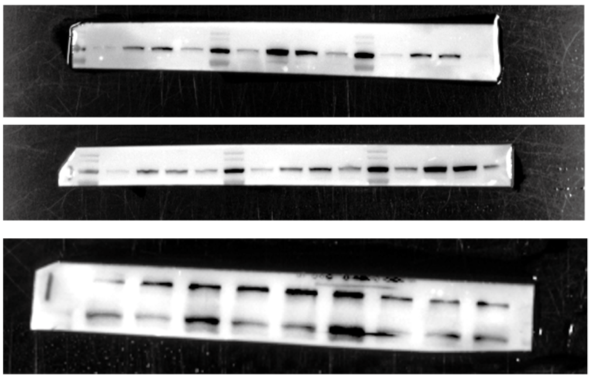

SLC7A11

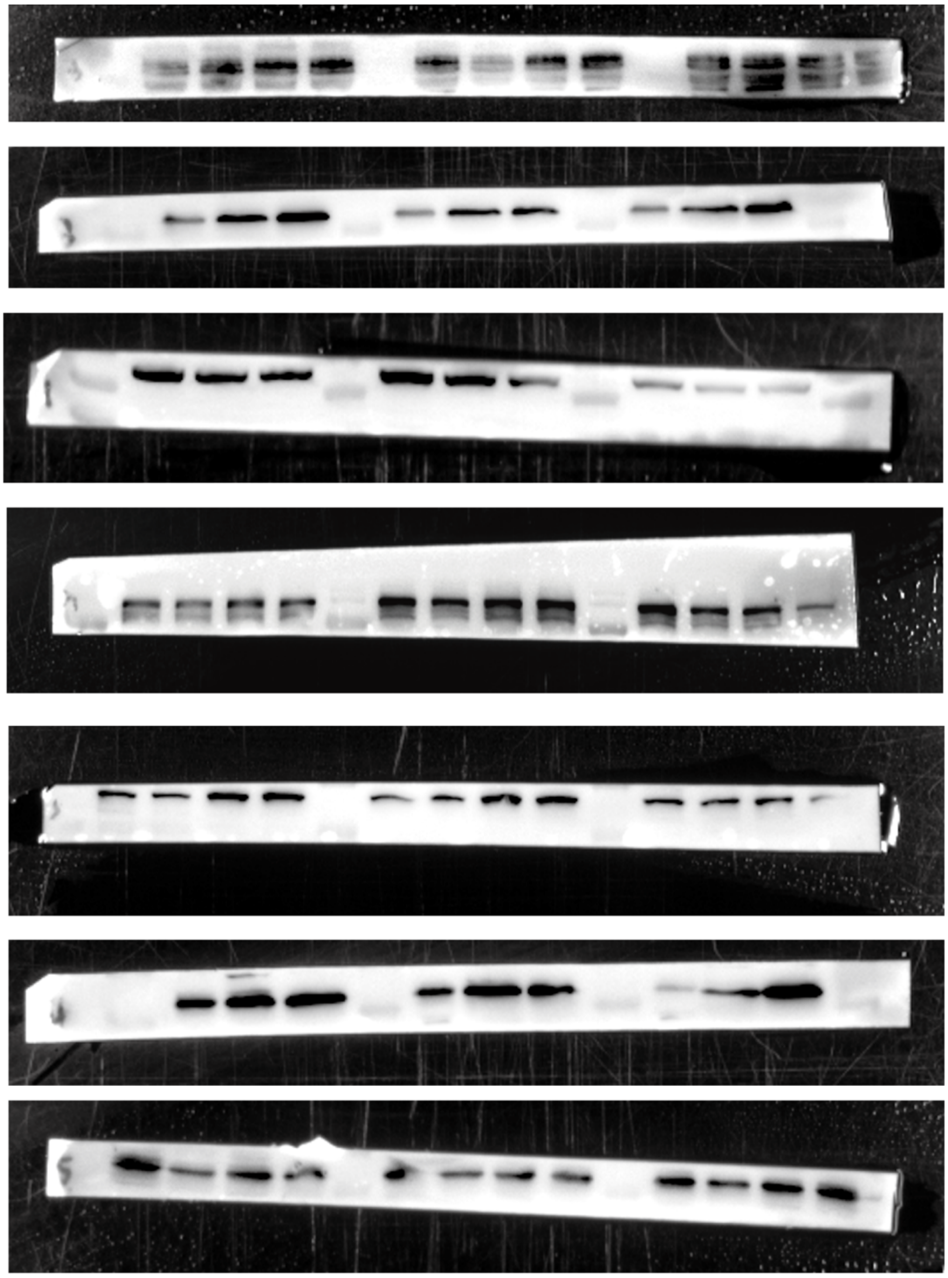

GPX4

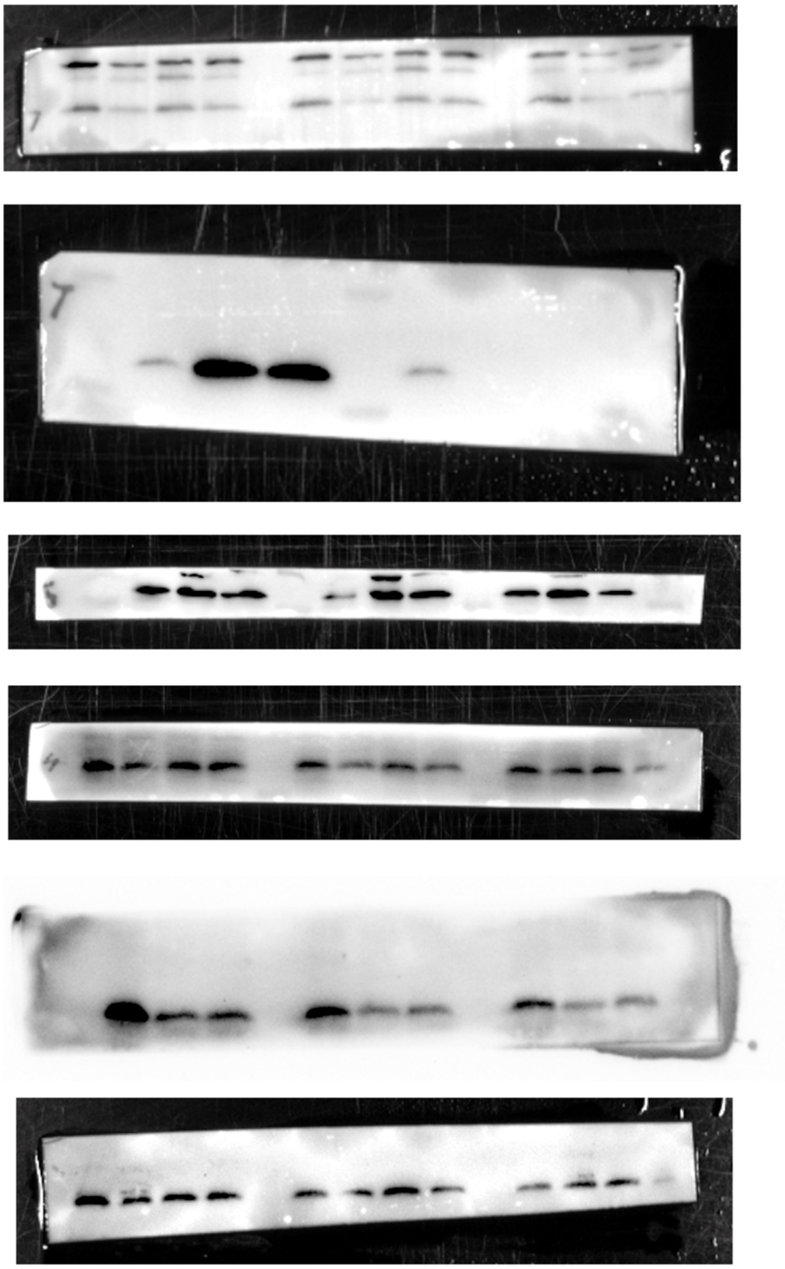

ATF3

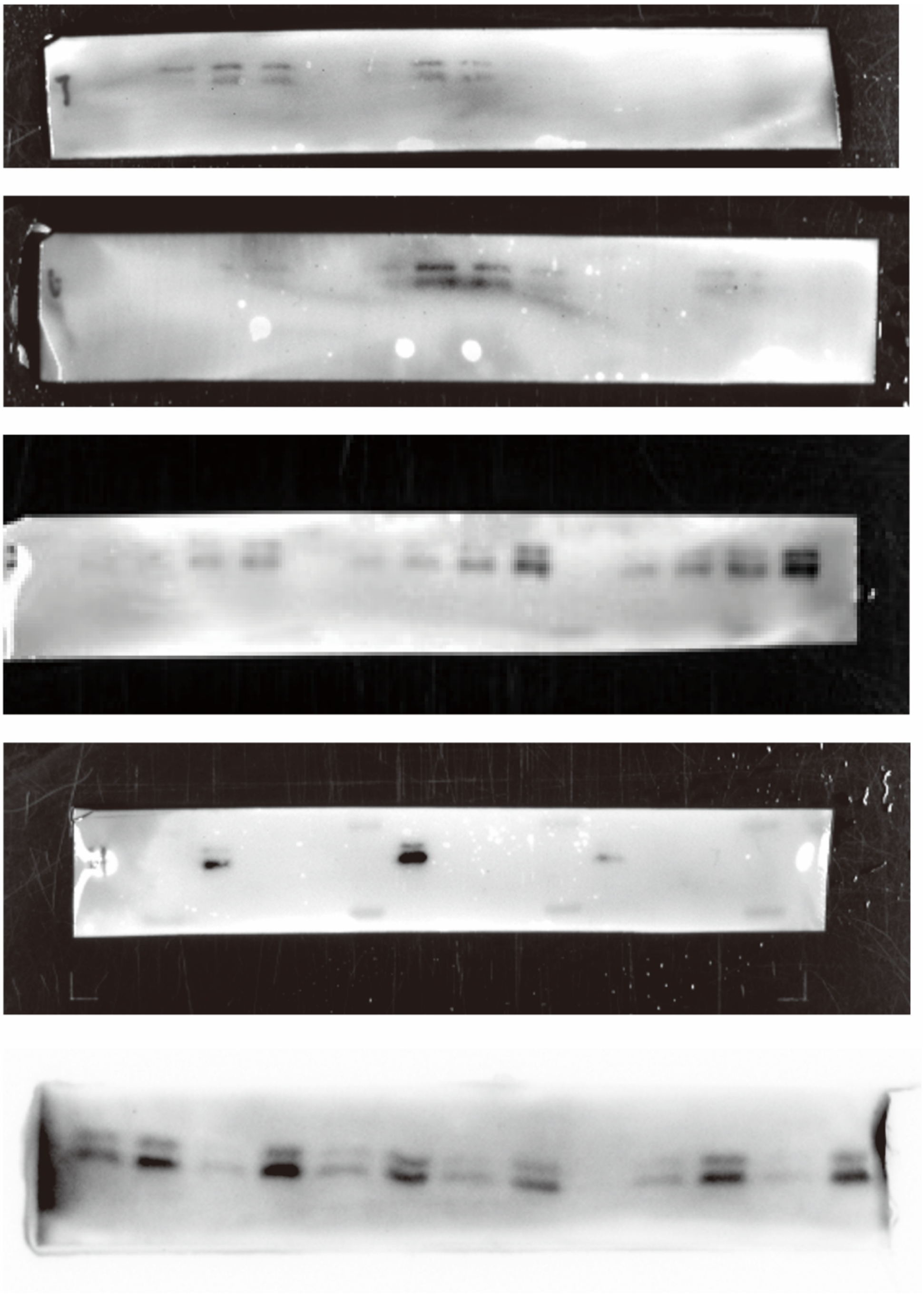

Actin

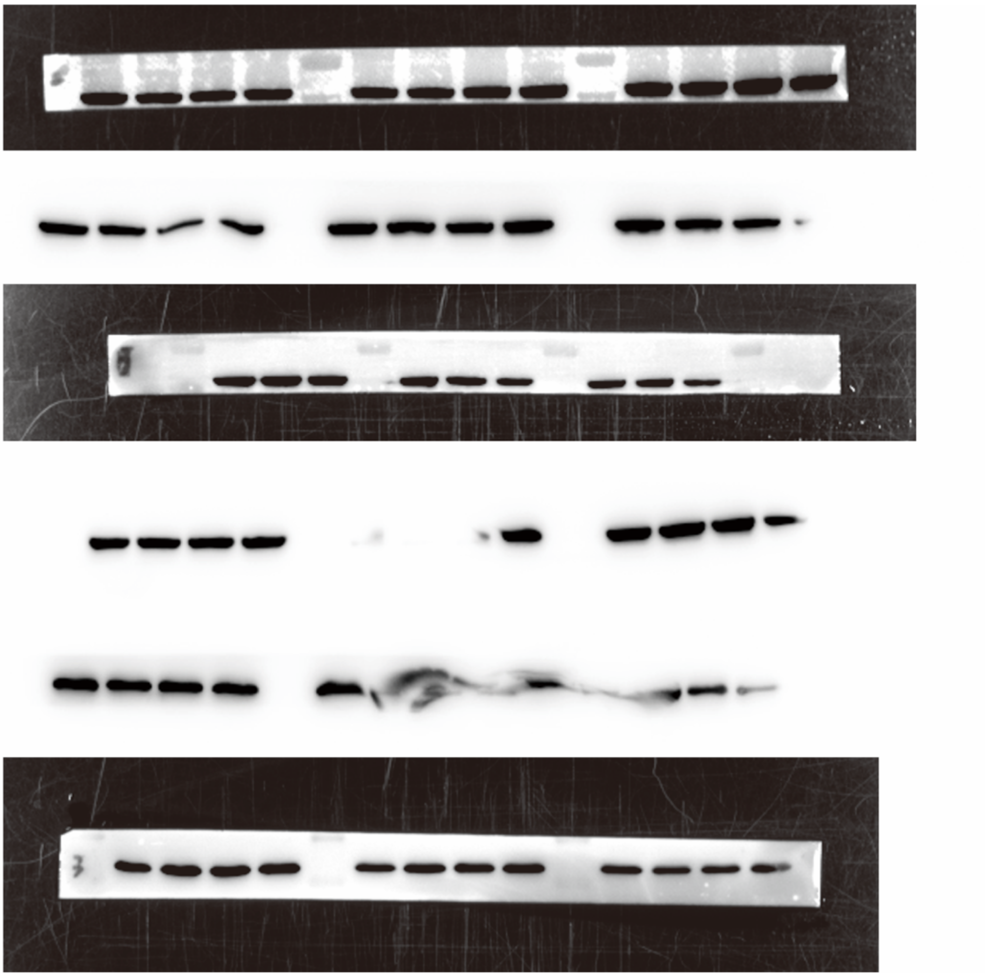

GAPDH

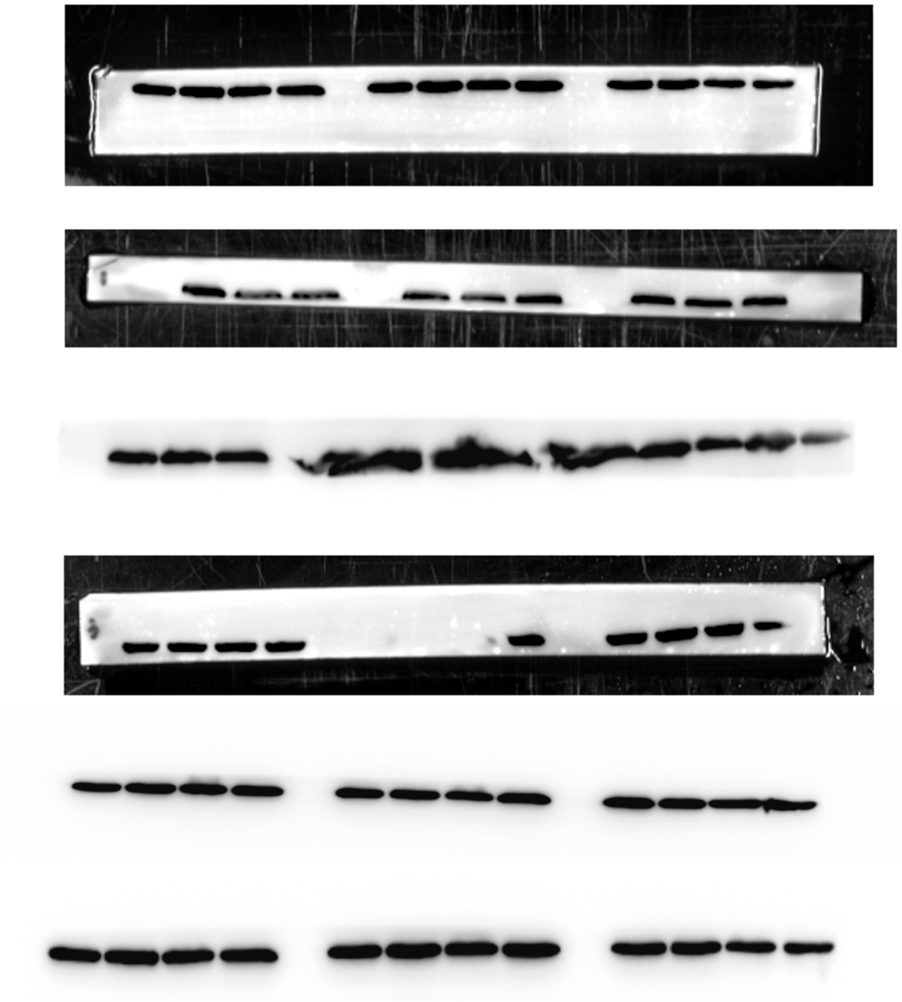

Supplement: Supplementary file 1 [file ijms-27-04373-s001.zip › ijms-4187387-supplementary.pdf]
